# Implementing a novel movement-based approach to inferring parturition and neonate caribou calf survival

**DOI:** 10.1371/journal.pone.0192204

**Published:** 2018-02-21

**Authors:** Maegwin Bonar, E. Hance Ellington, Keith P. Lewis, Eric Vander Wal

**Affiliations:** 1 Department of Biology, Memorial University of Newfoundland, St. John’s, Newfoundland and Labrador, Canada; 2 School of Environment and Natural Resources, Ohio State University, Columbus, Ohio, United States of America; 3 Department of Fisheries and Oceans, St. John’s, Newfoundland and Labrador, Canada; University of Western Ontario, CANADA

## Abstract

In ungulates, parturition is correlated with a reduction in movement rate. With advances in movement-based technologies comes an opportunity to develop new techniques to assess reproduction in wild ungulates that are less invasive and reduce biases. DeMars et al. (2013, Ecology and Evolution 3:4149–4160) proposed two promising new methods (individual- and population-based; the DeMars model) that use GPS inter-fix step length of adult female caribou (*Rangifer tarandus caribou*) to infer parturition and neonate survival. Our objective was to apply the DeMars model to caribou populations that may violate model assumptions for retrospective analysis of parturition and calf survival. We extended the use of the DeMars model after assigning parturition and calf mortality status by examining herd-wide distributions of parturition date, calf mortality date, and survival. We used the DeMars model to estimate parturition and calf mortality events and compared them with the known parturition and calf mortality events from collared adult females (*n* = 19). We also used the DeMars model to estimate parturition and calf mortality events for collared female caribou with unknown parturition and calf mortality events (*n* = 43) and instead derived herd-wide estimates of calf survival as well as distributions of parturition and calf mortality dates and compared them to herd-wide estimates generated from calves fitted with VHF collars (*n* = 134). For our data, the individual-based method was effective at predicting calf mortality, but was not effective at predicting parturition. The population-based method was more effective at predicting parturition but was not effective at predicting calf mortality. At the herd-level, the predicted distributions of parturition date from both methods differed from each other and from the distribution derived from the parturition dates of VHF-collared calves (log-ranked test: χ^2^ = 40.5, df = 2, *p* < 0.01). The predicted distributions of calf mortality dates from both methods were similar to the observed distribution derived from VHF-collared calves. Both methods underestimated herd-wide calf survival based on VHF-collared calves, however, a combination of the individual- and population-based methods produced herd-wide survival estimates similar to estimates generated from collared calves. The limitations we experienced when applying the DeMars model could result from the shortcomings in our data violating model assumptions. However despite the differences in our caribou systems, with proper validation techniques the framework in the DeMars model is sufficient to make inferences on parturition and calf mortality.

## Introduction

Significant life history events correspond with a change in movement behavior in wildlife species. For example, parturition in ungulates is generally associated with a steep reduction in movement rate [1] and movement rate slowly increases as offspring become more mobile. Due to recent advances in statistical techniques and GPS technologies [2,3], researchers have been able to not only estimate the timing of parturition events using movement data [4–7] but also to assess calf survival based on the movements of adult female caribou [8]. However, the transferability of novel methods may be limited by assumptions from the system in which the model was built. Recently, a promising advance in estimating parturition and neonate calf survival using movement data of adult females was developed for sedentary caribou (*Rangifer tarandus caribou*) herds in central British Columbia, Canada by DeMars et al. [8] (hereafter “the DeMars model”). Across their circumpolar distribution caribou exhibit variation in their movement behaviors, which may differ from those, exhibited in central British Columbia.

Previous methods for assessing reproduction in wild ungulates have included herd composition surveys (HCS) during the calving season [9], serum progesterone tests on captured animals [10], and vaginal implant transmitters [11]. Estimates of herd-wide calf survival can be made using recruitment rates from HCS [12] or from survival analysis using telemetry. These data collection methods can be invasive and prone to biases; particularly, non-representative sampling biases from telemetry and unequal detectability biases from the HCS [13]. DeMars et al. [8] proposed both an individual-based (IBM) and population-based (PBM) method for determining parturition and calf mortality events using adult movement data. The IBM uses movement models of GPS inter-fix distances (i.e., step length) of adult females and maximum likelihood estimation to infer parturition and calf mortality events [8]. The PBM uses a sample of adults with known parturition and calf mortality events to generate population level parturition and mortality movement thresholds based on inter-fix step length, which is subsequently used to identify the occurrence of parturition and calf mortality events in the larger adult GPS telemetry dataset [8]. Both methods are less invasive to neonate calves and have the potential to be more cost-effective than traditional methods as they rely solely on adult GPS telemetry data. Moreover, the DeMars model permits retrospective analysis to assess vital statistics (e.g., parturition and calf survival), increasing the value of previously collected GPS telemetry data. Despite these benefits, few studies have inferred parturition and survival in neonatal ungulates using the DeMars model (but see [14]). Furthermore, the model has not been independently validated.

Mirroring global caribou (*Rangifer tarandus*) declines [15], woodland caribou (*R*. *t*. *caribou*) population abundance has declined by more than 60% in the last 10 years in Newfoundland, which led the Committee on the Status of Endangered Wildlife in Canada to designate the caribou populations in Newfoundland as “Special Concern” [16,17]. In many ungulate systems including Newfoundland calf mortality is often the basis for initial population decline [18]. Given the recent population decline, woodland caribou demographics have been extensively monitored in Newfoundland. This presents an opportunity to retrospectively apply the DeMars model in this system. DeMars et al [8] outline two key assumptions regarding their model: 1) the assumption of movement independence among females and 2) the assumption of data quality. The caribou herds DeMars et al [8] used to build their model are considered sedentary in that they do not make long-distance migrations, and are assumed to move independently of one another and are not subject to group dynamics. When using their methods, DeMars et al [8] indicate that for data sets with fix success rates of <90%, estimates of offspring survival may be unreliable and thus assumes that data quality is sufficiently high to make accurate model inferences.

Our aim is to apply the DeMars model to two caribou populations in Newfoundland, Canada, where mother-offspring data were available and movement behaviors of caribou might violate the assumptions of the DeMars model. The purpose of our study was two-fold: 1) apply these two novel methods to different ungulate populations and study systems that may violate model assumptions to determine if inferences are possible; and 2) examine the accuracy of our data to generate herd-wide survival estimates and distributions of parturition and calf mortality dates. We expect the DeMars model will be transferable in at least some capacity for both test populations of caribou in Newfoundland. However, the DeMars model will likely make more accurate inferences when applied to the more sedentary of the two populations that are similar in behaviour to the herds DeMars et al [8] used to build their model.

## Materials and methods

### Study area

We conducted our study in Newfoundland, a 108 860 km^2^ island in eastern Canada (47°44 N, 59°28 W to 51°44 N, 52°38 W), with a humid–continental climate and ample year-round precipitation. The landscape consisted of coniferous and mixed forests of balsam fir (*Abies balsamea*), black spruce (*Picea mariana*) and white birch (*Betula papyrifera*), as well as bogs, lakes, and barren rock. Our analysis focused on caribou in two separate herds: Middle Ridge and Fogo Island. Middle Ridge is located on the south central portion of Newfoundland and Fogo Island (237 km^2^) is situated off the northeastern coast of Newfoundland. The landscape that these two herds occupy is broadly similar, however, Fogo Island is separated from mainland Newfoundland by approximately 12 km and the Fogo Island herd is sedentary and does not display the same migration pattern as the Middle Ridge herd.

### Overview

**Fig 1:**
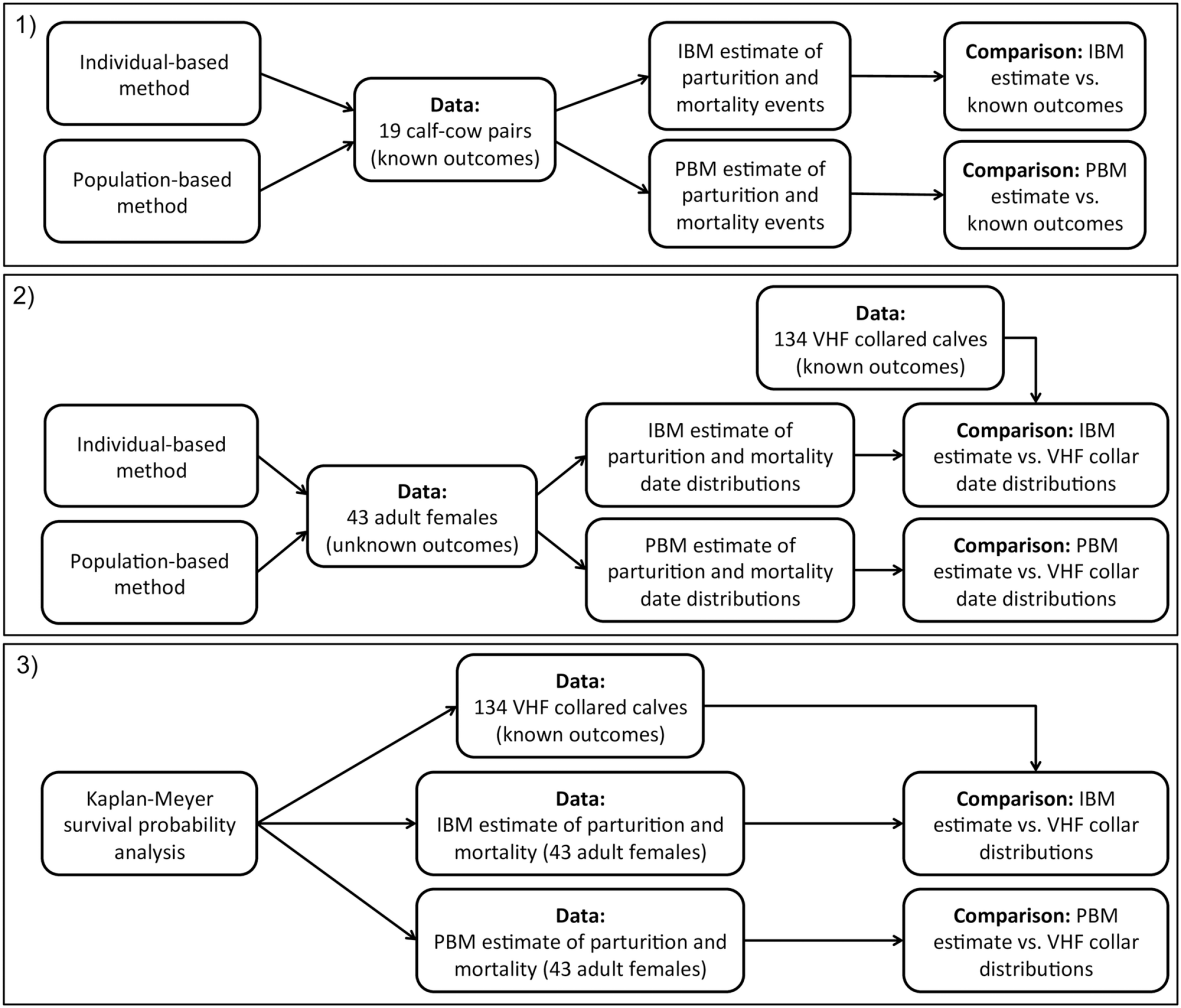
Overview of the DeMars model application to two caribou populations in Newfoundland, Canada. We applied the individual-based method and population-based method in three different ways. 1) We estimated parturition and calf mortality events using the two methods to adults (*n* = 19) with known parturition and calf mortality events from two different woodland caribou herds in Newfoundland. 2) We estimated herd-wide distributions of parturition and calf mortality dates using the two methods and a multi-year (*n* = 43) adult telemetry dataset and compared those distributions to the herd-wide distributions of parturition and calf mortality dates derived from concurrent calf telemetry data (*n* = 134). 3) We generated herd-wide calf survival estimates from Kaplan-Meyer probability curves using the two methods and a multi-year (*n* = 43) adult telemetry dataset and compared those probability curves to herd-wide survival probability curves derived from concurrent calf telemetry data (*n* = 134).

First, we compared estimates of parturition and calf mortality events generated using the DeMars individual based model (IBM) and population based model (PBM) to adults (*n* = 19) with known parturition and calf mortality events from two different woodland caribou herds in Newfoundland. Second, we compared herd-wide calf survival estimates and distributions of parturition and calf mortality dates using both the DeMars IBM and PBM from a large multi-year (*n* = 43) adult telemetry dataset to the herd-wide survival estimates and distributions of parturition and calf mortality dates derived from concurrent calf telemetry data (*n* = 134). This required 1) GPS telemetry data from adult female caribou with known and unknown parturition and calf mortality events; 2) herd-wide estimates of calf survival; 3) herd-wide estimates of the distribution of parturition date; and 4) calf mortality dates from collared calves (Fig 1).

### Caribou telemetry and observational data

Adult female caribou were captured using a net gun or darted using the immobilizing agent Carfentanil. Females were not captured or immobilized during the calving season to avoid transferring immobilizing drugs to calves. GPS 4400M collars (1240g, Lotek Wireless Inc., Newmarket, ON, Canada) were deployed on 43 adult females in the Middle Ridge (MR: 2009–2013) herd and 9 adult females in the Fogo Island (Fogo: 2015) herd. Sampling adults followed typical large mammal procedures, i.e., haphazard or convenient sampling. A GPS fix was attempted every two hours from May 21 –July 31 and every five hours for the remainder of the year for females in the MR herd and every two hours year-round for females in the Fogo herd. The status of parturition and calf mortality was known for all 9 collared adult females in the Fogo herd (using direct observation; see below) and for 10 of the collared adult females in the MR herd (via paired VHF-collared calves; see below). Pregnancy status was visually determined for the 19 adult females upon capture.

From 2009–2013, caribou calves from the MR herd were located from helicopter and captured on foot during the calving season. Most calves were captured <5 days after birth. Ear-tags and expandable 200g VHF radio-collars (Telemetry Solutions, Concord, CA, USA) were deployed on 134 calves in the MR herd; this included 10 calves that were paired with GPS-collared adult females in 2009. These collars were under the recommended 5% of the individual’s body mass [19]. Calves were visually relocated by helicopter within 24 hours of initial capture to ensure they had re-bonded with their mothers. Passive transfer status was not determined for each calf upon capture. Survival was monitored daily during the first week of post capture, and then at least twice a week through August. When a neonatal mortality signal was detected, the collar was located aerially and field crews located calf remains and assigned cause of death based on remains and site conditions (see [20] for full details).

We assessed caribou parturition and calf survival for the Fogo herd in 2016 through visual observation. We located each collared adult female on foot every week (*x* = 7; range = 1–19 days) from 24 May 2016 until 30 June 2016 and then every three weeks (*x* = 25; range = 17–34 days) from 1 July 2016 until 30 July 2016. We located each female at least three times and we confirmed that a calving event occurred when a female was observed with a calf. We continued locating the adult females after calving to assess calf survival until four weeks of age. We continued to track and observe adult females after calf loss was suspected to confirm calf status. As none of these females were subsequently observed with a calf, we assumed that the true status of the calf was known Memorial University of Newfoundland Animal Care and Use Committee approved this study (16-03-EV).

### Estimating parturition and calf mortality events

#### DeMars individual-based method

**Fig 2:**
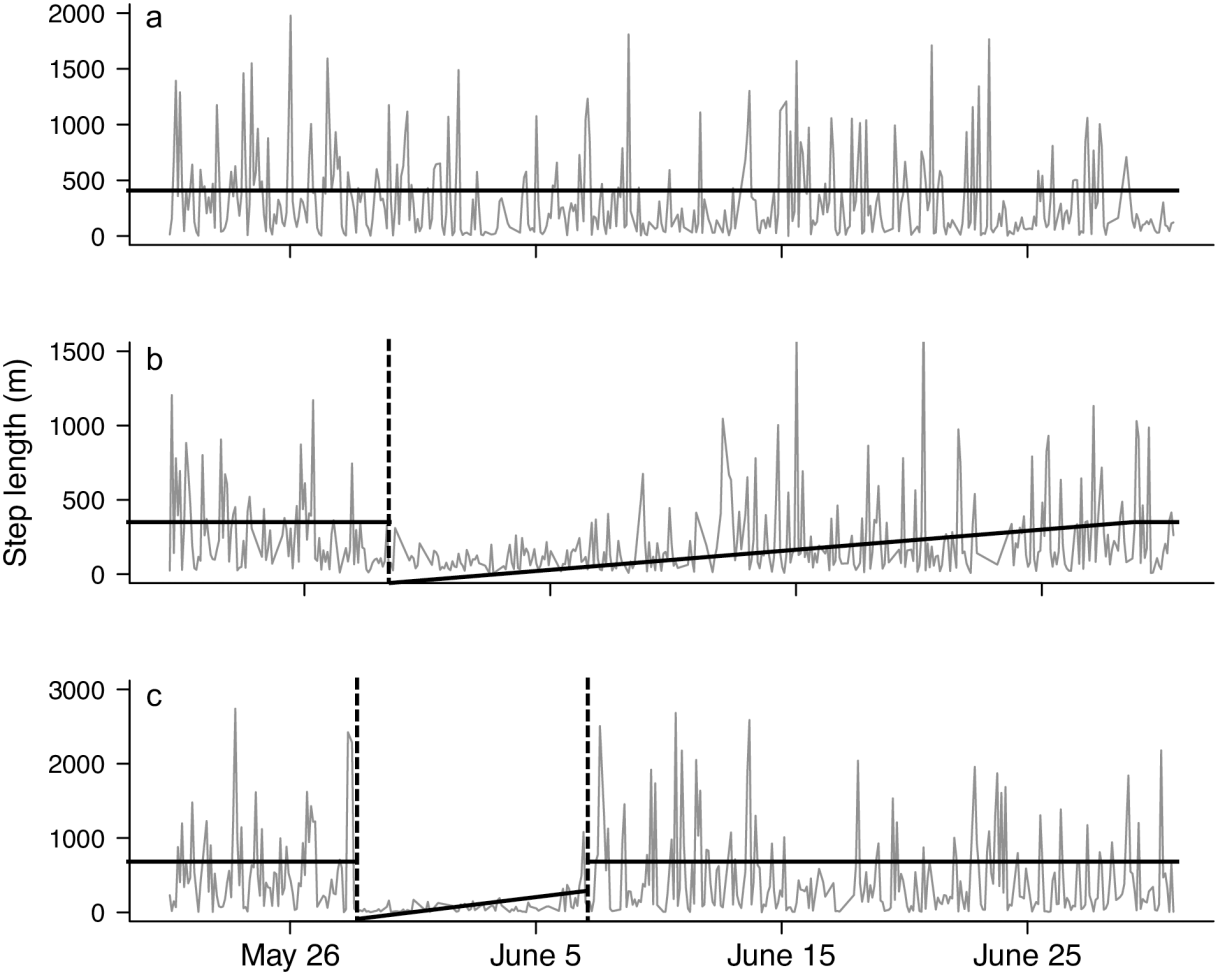
Examples of *a priori* movement models used in an individual-based method to infer parturition and calf mortality events in female woodland caribou (*Rangifer tarandus caribou*) (sensu 8) and actual movement characteristics of female caribou. Gray line indicates the movement pattern of female. Black line indicates the predicted mean step length from movement model. The movement data used to generate the movement patterns were collected from either Fogo Island herd (a and b) or Middle Ridge herd (c). (a) No parturition event occurred, this is indicated by no break point in the mean movement rate. (b) Parturition event occurred on approximately 29 May and no mortality event occurred (prior to four weeks of age), this is indicated by a single break point in mean movement rate followed by a gradual increase in movement rate until mean movement rate reaches that of pre-parturition (at approximately four weeks post-parturition). (c) Parturition event occurred on approximately 28 May then a calf mortality event occurred on approximately 6 June, this is indicated by two break points in mean movement rate, one at the point of parturition and subsequently when the female lost her calf before four weeks and immediately returns to pre-parturition movement rate.

Following DeMars et al. [8], our IBM for parturition and calf mortality events used three *a priori* models representing the three possible states of a female ungulate during calving season: 1) no parturition; 2) calf survived to four weeks old; and 3) calf mortality occurred before the calf was four weeks old (Fig 2). In the model, “no parturition”, the mean step length remained constant over time. In the other two models, an event (i.e., parturition or calf mortality) was represented by an abrupt change in the mean step length: a decrease in mean step length represented parturition and an increase represented calf mortality. Thus, in the model, “calf survived to four weeks old”, mean step length dropped abruptly, creating a breakpoint at calving, followed by an increase in mean step length with a slope equal to the ratio between the scale parameter and the number of step lengths required for the calf to reach adult movement rates. Conversely, in the model, “calf mortality occurred before four weeks old”, mean step length dropped abruptly, creating a break point at parturition, followed by an increase in mean step length with a slope equal to the ratio between the scale parameter and the number of step lengths required for the calf to reach adult rates of movement. This slope, however, was interrupted by an abrupt increase in mean step length to the original mean step length of the adult female at the point of calf mortality [8]. All three of the *a priori* models assumed that step length was exponentially distributed and should differ only in the scale parameter (i.e., mean step length). Calf status was assessed up to four weeks as calf mobility after four weeks begins to approach adult movement rates [8].

We screened and removed any erroneous data points arising from unrealistic movement patterns following Bjørnerass et al. [21]. We globally removed 6% of fixes from MR and 10% from Fogo. We further rarefied the datasets to exclude the top 1% of step lengths for each individual, which are thought to be associated with calf capture or predator avoidance behavior [8]. After rarefication, the mean per-collar fix rate (number of successful fixes per number of attempts; [22]) was 80% (range: 53–93%). MR appeared to have a higher mean per-collar fix rate (90%, range: 85–93%) than Fogo (80%, range: 53–87%). Following DeMars et al. [8], we used only step lengths calculated from successive GPS fixes. We used a visual examination of the distributions of step lengths for all 19 calf-cow pairs to determine that the step lengths were exponentially distributed (S1 Appendix). We then generated the IBM for the 10 adult females with known calf status from the MR herd in 2009 and the 9 adult females with known calf status from the Fogo herd in 2016. We present graphical representation of step lengths for all 19 adult females with known calf status in S1 Appendix. We then fit the data to each *a priori* model and determined the most supported model using Akaike’s Information Criterion (AIC; [23]). We tested our IBM over the time interval 21 May– 30 July for 43 individuals in the MR herd and 8 individuals in the Fogo herd. We tested our IBM over the time interval 30 May– 30 August for one individual to account for a record-late birth in the Fogo herd in 2016 [24]. After applying the IBM to our data, we then compared the predicted calf survival outcomes generated from the IBM with the known outcomes for each female.

#### DeMars population-based method

The PBM used population level event thresholds (parturition and calf mortality) of 3-day average movement rates (TDAM) in a 3-day moving window analysis to predict calving and calf survival events. Following DeMars et al. [8], to define the parturition threshold, we first generated a distribution of TDAM rates for 3 days post-calving for females who had calves survive to 1 week. We then converted the distribution of movement rates to a kernel density estimate (KDE), which represented the population-level distribution of TDAM rates 3 days post-calving. We transformed this KDE into a cumulative distribution function (CDF) that represented the proportion of the population expected to move at or below this threshold. We then took the 99.9% quantile of the CDF as the parturition threshold; we assumed that movement below this threshold during the moving window analysis indicated calving [8]. To more accurately reflect the true 3-day post-parturition window and thus improve the biological accuracy of the parturition threshold, we estimated parturition date as 1 day prior to calf captures. Capture date, 3 days prior, and 2 days prior were also tested but were less accurate overall (data not shown).

To generate the calf mortality threshold from a distribution of TDAM rates we followed the same methods outlined above that were used to generate the parturition threshold, this time for 2–4 weeks post-parturition for females who had calves survive to 4 weeks old. The 99.9% quantile of CDF from this data represented the maximum TDAM rate of a female with a calf up to four weeks old (i.e., calf mortality threshold); we assumed that movement above this rate indicated calf mortality [8].

Prior to calculating the parturition and calf mortality thresholds, we rarefied the data to exclude the top 1% of step lengths. We assumed this removed any step lengths that could have been associated with calf capture or predator avoidance [8]. We generated the parturition and calf mortality thresholds (in the manner described above) in program R [25] using a function provided by DeMars et al. [8]. We modified the function used to generate the parturition thresholds to reflect the variation in TDAM rates within our data (S2 Appendix).

We generated PBM estimates for each herd (MR, Fogo) and the combined herds using the 19 adult females with known parturition and calf mortality events. The PBM required a subset of the population that had experienced both parturition but not calf mortality to generate event thresholds—there were 10 adult females that fit this description (7 in MR and 3 in Fogo). To introduce stochasticity and prevent sampling bias, we iteratively sampled all possible combinations of 5 from the 9 adult females across both herds (*n* = 126) and all possible combinations of 4 from the 7 adult females in MR (*n* = 35; i.e., k-fold) for generating event thresholds. The iterative sampling technique was not possible for the Fogo herd as only 3 of the 9 collared females could be used to calculate the calving and calf loss thresholds. This included the female with the record-late birth outside of the calving season [24], which we chose to exclude since the female may not be representative of the whole herd. Therefore, we had only one estimate of event thresholds for the Fogo herd generated using 2 out of 9 collared females. Using these event thresholds, we then compared the PBM-based predictions of parturition and calf mortality events to the known status of all 19 adult females across both herds and for the MR and Fogo herds separately. We considered the prediction conclusive when the proportion of occurrence was ≥ 0.8 otherwise the prediction was inconclusive.

#### Estimating herd-wide survival, parturition, and mortality date distributions

To generate herd-wide estimates of survival and distributions of parturition and calf mortality dates, we applied both the DeMars IBM and PBM to the 43 GPS-collared adult females from MR. We generated event thresholds required for the PBM using the 7 adult females from the MR herd that had experienced both parturition and calf mortality events. We generated density distributions of the estimated parturition dates and mortality dates derived from the IBM and the PBM. We also converted the estimated calf parturition and mortality events from the IBM and PBM into Kaplan-Meier survival probability curves using the survival package [26] in R. Following Ellington et al. [13], we generated herd-wide survival curves and parturition and mortality date distributions from the 134 VHF-collared calves from the MR herd and compared them to survival curve and distributions generated using the DeMars IBM and the PBM. The VHF collaring date was used as a proxy for parturition dates. Calves were collared during a 1–3 day period at the suspected peak of calving season on any given year. In all analyses, we generated IBM and PBM models using both a 2-hour GPS fix time interval dataset and a rarified 4-hour GPS fix time interval dataset (to reproduce the methods used by DeMars et al. [8]).

## Results

We found that the predictions from the DeMars model for 2-hour time interval were more accurate compared to the 4-hour time interval and have chosen to present the overall results based on the 2-hour time series. The 4-hour time series can be found in S3 Appendix.

### Estimating parturition and calf mortality events

#### DeMars individual-based method

The DeMars IBM failed to definitively distinguish (i.e., ΔAIC > 2 for the most parsimonious model) a parturition and calf mortality status for 2 out of 9 adult females from the Fogo herd and definitively distinguished parturition and calf mortality status for the remaining 7 adult females. In both cases where the IBM failed to distinguish the most parsimonious model, the models “calf survived to four weeks old” and “calf mortality occurred before calf was four weeks old” were competing. In one case, parturition occurred, and the calf survived to four weeks (ΔAIC = 1.23), and in the other case parturition occurred and calf mortality occurred before four weeks (ΔAIC = 1.94). In these cases we considered the IBM method successful in predicting parturition but inconclusive in predicting mortality events. The DeMars IBM definitively distinguished (though not always correctly) a parturition and calf mortality status for all 10 adult females from the MR herd.

The IBM correctly classified the two adult females who had no parturition event. The IBM also correctly predicted parturition in 7 of 17 adult female caribou in which parturition occurred (4 of 10 for MR and 3 of 7 for Fogo; Table 1). In situations in which parturition occurred but the IBM method failed to predict parturition (*n* = 10), calf mortality did not occur in 8 of 10 cases (6 of 6 in MR and 2 of 4 in Fogo; Table 1). Indeed, the IBM method correctly identified only 1 of 10 adult females in which parturition occurred but calf mortality did not occur (1 of 7 in MR and 0 of 3 in Fogo; Table 1). The IBM method predicted calf mortality in 4 of 7 adult female caribou in which calf mortality occurred (3 of 3 for MR and 1 of 4 for Fogo; Table 1).

**Table 1:** Parturition and calf mortality status predictions derived from the individual-based method (IBM) from DeMars et al. [8]. Predictions are for 19 calf-cow pairs from Middle Ridge and Fogo Island herd for which calf status was known.

Herd	Status	Observed	IBM Predicted
Middle Ridge	Parturition	10	4
	No Parturition	0	6
	Calf Survival	7	1
	Calf Mortality	3	3
Fogo Island^1^	Parturition	7	3
	No Parturition	2	6
	Calf Survival	3	0
	Calf Mortality	4	1

^1^ The individual based method for predicting parturition and calf mortality status was inconclusive (competing models) for two adult females.

#### DeMars population-based method

The event thresholds using the PBM were higher in the MR herd than the Fogo herd (parturition: 208 m/hr [range: 146–266 m/hr] vs 23 m/hr and calf mortality: 407 m/hr [range: 217–567 m/hr] vs 126 m/hr). Perhaps this is not surprising given that the dimensions of the island confine space use patterns of caribou in the Fogo herd. Surprisingly, the event thresholds using the combined MR and Fogo data were higher than the MR event thresholds, perhaps due to larger sample size within the k-fold subset (parturition: 259 m/hr [range: 180–296 m/hr] and calf mortality: 460 m/hr [range: 210–563 m/hr]).

In general, the PBM performed better for each herd when it used herd-specific event thresholds than when it used event thresholds derived from the combined herds (S1 Appendix), thus we focus our results on PBM based on herd-specific event thresholds. Because of the iterative process in generating event thresholds for the MR herd, multiple outcomes were generated for each event (parturition, no parturition, calf mortality, calf survival). The resulting estimates for each event were pooled as proportion of occurrence across all the event thresholds. Due to small sample size, there was no iterative process in generating event thresholds for the Fogo herd, thus there were no inconclusive predictions.

The PBM correctly predicted parturition status for 16 of 17 females across both herds (15 of 15 parturient and 1 of 2 non-parturient). For one female in the Fogo herd it predicted parturition when parturition did not occur (Table 2). The PBM did not predict calf mortality correctly and conclusively; in cases where calf mortality occurred the PBM predicted no calf mortality for 2 out of 4 individuals in the Fogo herd (Table 2) and was inconclusive for all individuals (*n* = 3) in the MR herd (Table 2). The PBM predicted calf mortality did not occur in 6 of 8 females where calf mortality did not occur (5 of 7 in MR and 1 of 1 in Fogo; Table 2).

**Table 2:** Parturition and calf mortality status predictions derived from the population-based method (PBM) from DeMars et al. [8] for 19 calf-cow pairs from Middle Ridge and Fogo Island herd for which calf status was known. Predictions for Middle Ridge herd were generated by iteratively sampling of 4 out of the 7 females that could be used to generate parturition and calf mortality thresholds for the model and tested the on the remaining 6 individuals for all possible combinations. Predictions were pooled and the proportion of each prediction was calculated for every individual. Predictions for Fogo Island herd were generated by using 2 females that could be used to generate parturition and calf mortality thresholds for the model and tested the on the remaining 7 individuals, thus an iterative process was not possible and there is only one estimate for each event. The 2 individuals used to generate the event thresholds were not included in testing.

	Known Status		PBM Predictions (proportion of time each status was predicted)			
ID^1^	Parturition	Calf Survival^2^	Parturition	No Parturition	Calf Mortality^2^	Calf Survived^2^
MR2009a01	Parturition	Survived	1.00	0.00	1.00	0.00
MR2009a04	Parturition	Survived	1.00	0.00	0.00	1.00
MR2009a06	Parturition	Mortality	1.00	0.00	0.49	0.51
MR2009a07	Parturition	Mortality	1.00	0.00	0.46	0.54
MR2009a08	Parturition	Survived	1.00	0.00	0.07	0.93
MR2009a09	Parturition	Survived	1.00	0.00	0.40	0.60
MR2009a16	Parturition	Survived	1.00	0.00	0.00	1.00
MR2009a25	Parturition	Survived	1.00	0.00	0.20	0.80
MR2009a26	Parturition	Survived	1.00	0.00	0.00	1.00
MR2009a27	Parturition	Mortality	1.00	0.00	0.46	0.54
FO2016002	Parturition	Mortality	TRUE	FALSE	TRUE	FALSE
FO2016005	No Parturition	NA	FALSE	TRUE	NA	NA
FO2016010	Parturition	Mortality	TRUE	FALSE	FALSE	TRUE
FO2016011	Parturition	Survived	TRUE	FALSE	FALSE	TRUE
FO2016012	Parturition	Mortality	TRUE	FALSE	TRUE	FALSE
FO2016014	Parturition	Mortality	TRUE	FALSE	FALSE	TRUE
FO2016015	No Parturition	NA	TRUE	FALSE	TRUE	FALSE

^1^Individual IDs beginning with MR are from Middle Ridge herd and individual IDs beginning with FO are from Fogo Island herd.

^2^ When parturition did not occur there was no calf mortality status and when parturition was not predicted there was no calf mortality status predicted.

#### Herd-wide survival estimates and distributions of parturition and mortality dates

**Fig 3:**
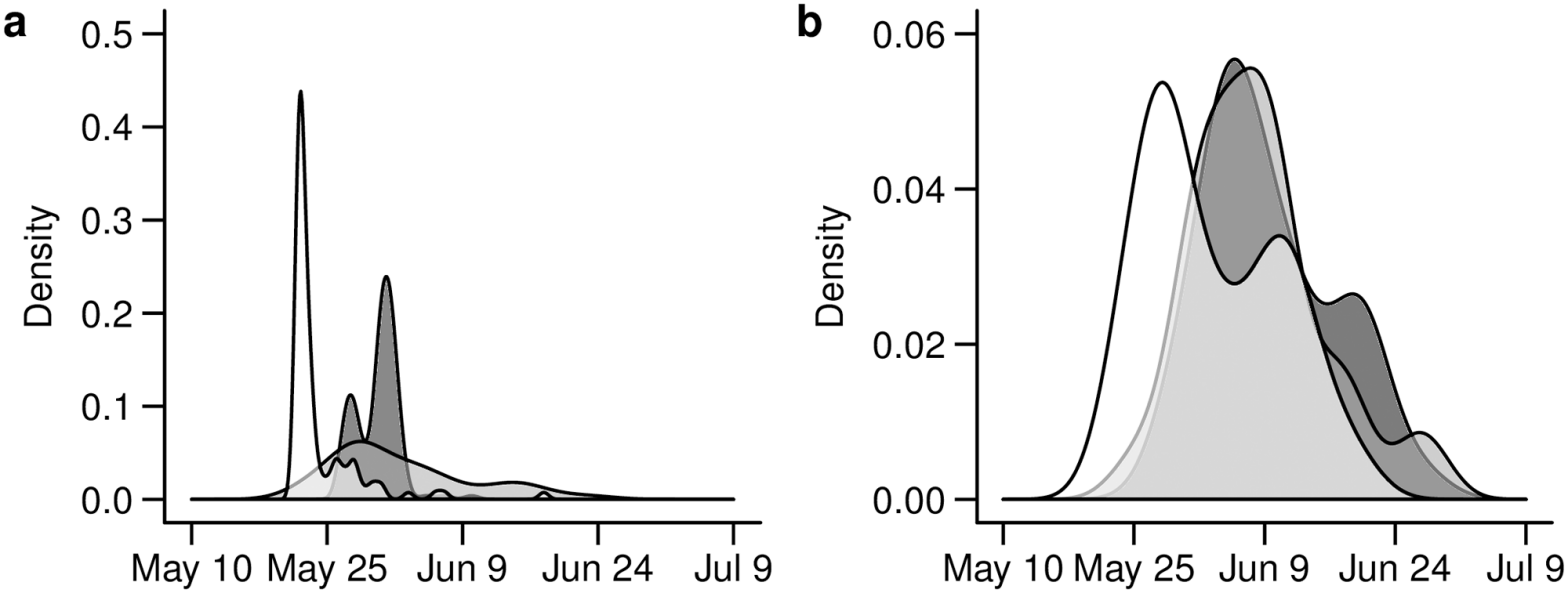
Comparison of density distributions of herd-wide VHF calf collaring dates and mortality dates from 134 calves from Middle Ridge herd between 2009–2013 [13] with estimated parturition and calf mortality dates derived from DeMars et al. [8] individual-based method (IBM) and population-based method (PBM) for 43 adult females from Middle Ridge herd between 2009–2013. (a) Density distributions of herd-wide VHF calf collaring dates (dark gray), estimated parturition dates derived from IBM (light gray) and PBM (white). (b) Density distributions of herd-wide VHF calf mortality dates (dark gray), estimated calf mortality dates derived from IBM (light gray) and PBM (white).

The predicted distributions of parturition date from the IBM and PBM were different from each other and from the distribution derived from the VHF-collared calves. The IBM predicted that parturition occurred in a wide distribution with only a small peak occurring > 1 week before the observed peak from the VHF-collared calf data, which suggested a long, diffuse calving season (Fig 3a). Conversely, the PBM predicted that parturition occurred in a distribution with a steep peak > 2 weeks before the observed peak from the VHF-collared calf data, which suggested a calving season broadly similar to the observed calving season but with the majority of parturition events occurring much earlier than they have been observed (Fig 3a). Among individuals with known parturition events, the IBM predicted parturition dates were within 1 day of the collared date (*n* = 3; i.e., the IBM method when accurate was highly precise; S1 Appendix). The PBM method predicted parturition dates that were typically ≥ 6 days underestimated compared to collar date (*n* = 8; i.e., the PBM method was highly accurate but had a consistent bias; S1 Appendix)

The predicted distribution of calf mortality dates from the IBM and PBM were broadly similar to the observed distribution from the subset of VHF-collared calves in which mortality occurred prior to 4 weeks of age (Fig 3b). The only major discrepancy was that the peak in mortality date occurred slightly earlier using the PBM than the IBM or the observed VHF-collared calves (Fig 3b). Among individuals with known mortality events (*n* = 3), the IBM method identified all mortality events but predicted mortality dates varied (-3 to 10 days difference from actual mortality event; i.e., the IBM method was highly accurate but imprecise). The PBM method identified only two of the three known mortality events and consistently underestimated the mortality date (i.e., the PBM method was less accurate and also had a consistent bias).

**Fig 4:**
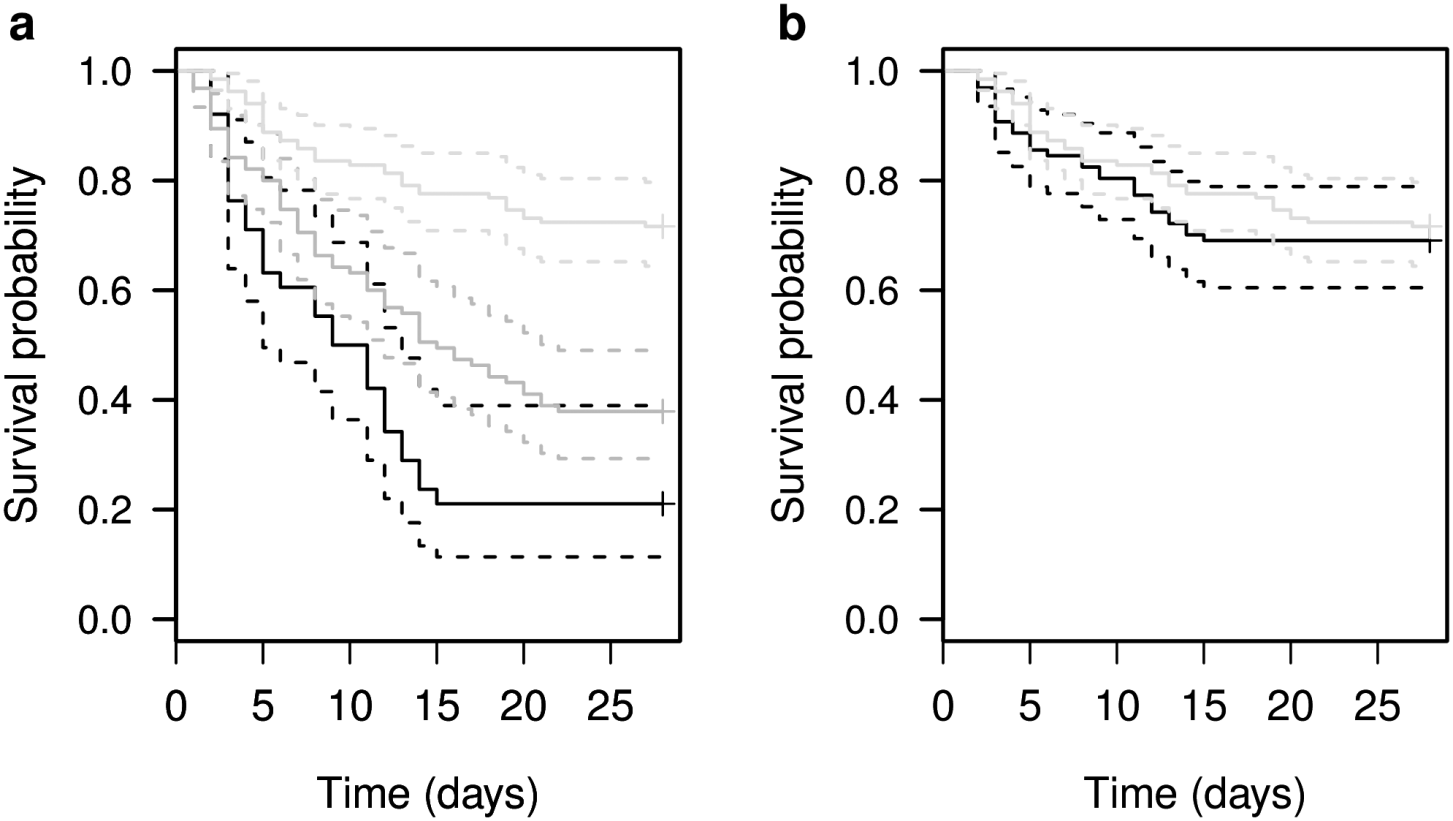
(a) Kaplan-Meier survival probability curves comparing survival data generated from 43 GPS-collared females in Middle Ridge from 2009–2013 using DeMars et al.’s [8] individual-based method (IBM; black), and population-based method (PBM; dark gray), and calf mortality from 134 VHF-collared calves in Middle Ridge from 2009–2013 (light gray). Dotted lines are 95% confidence intervals. (b) Kaplan-Meier survival probability curves comparing calf mortality data from 134 VHF-collared calves from Middle Ridge herd from 2009–2013 (light gray) to survival data generated by using a combination of the IBM and PBM models for 43 GPS-collared females in Middle Ridge herd from 2009–2013 (black). Parturition was determined for 43 GPS collared females from 2009–2013 using the PBM and then calf mortality was determined using the IBM. For calves whose parturition was predicted using the PBM, if the IBM did not predict calf mortality before four weeks we assumed the calf survived.

Both the DeMars IBM and the PBM estimated lower herd-wide survival than what was observed from VHF-collared calves (log-ranked test: χ^2^ = 40.5, df = 2, *p* < 0.01; Fig 4a). The IBM estimated 38 parturition events and 30 mortality events, and the PBM estimated 95 parturition events and 59 mortality events for the 43 females over the 5 years. Out of the 134 VHF-collared calves there were 38 mortality events. However, given the performance of the IBM and PBM when estimating parturition and calf mortality, we generated a survival curve where the PBM was used to identify parturition and the IBM was used to identify mortality (assuming the parturition status identified by PBM). The combined method estimated 97 parturition events and 30 mortality events. Survival rates that we estimated with this combined IBM and PBM method were not statistically different from the 134 VHF collared calves in MR from 2009–2013 (log-ranked test: χ^2^ = 3.9, df = 1, *p* = 0.05; Fig 4b).

## Discussion

In ungulates, parturition corresponds with an abrupt drop in movement rate [1,27]. This relationship has been used in the past to estimate parturition from movement data with mixed success [1,4,27,28]. DeMars et al. [8] took this relationship further and developed two methods (individual- and population-based; IBM, PBM) to identify neonate calf mortality in addition to parturition for sedentary caribou. We intended to see if the DeMars model will work to assign parturition and calf mortality statuses to adult female caribou for the purpose of survival analysis despite violating the assumptions. In general, the accuracy of both methods was lower for caribou in two herds in Newfoundland relative to what DeMars et al. [8] observed for caribou in British Columbia. Our IBM did not perform well at predicting parturition (particularly if calf mortality did not occur), but performed better at predicting calf mortality. Conversely, our PBM did not perform well at predicting calf mortality, but predicted parturition with near perfect accuracy. On their own, these methods did not generate accurate herd-wide survival estimates based on VHF-collared calves in Newfoundland. Combined, however, the two methods produced herd-wide survival estimates similar to radio-telemetry.

DeMars et al. [8] developed their original model using sedentary woodland caribou in British Columbia. The sedentary caribou ecotype tend to isolate themselves from other individuals to decrease detection from predators [29], whereas migratory caribou will space away from the distribution of predators and calve in large aggregations [30,31,32]. Thus, sedentary caribou may meet the assumption of independent movement for the DeMars model, but migratory caribou may not. Indeed, the poorer performance of both the IBM and PBM with the migratory caribou of the MR herd (relative to [8]) might be partially driven by violating the assumption of independent movement. Caribou in the MR herd move together to the calving grounds and most individuals arrive at the calving grounds within a few days of each other, even if parturition does not occur at this time. This behavior could have led the PBM to consistently underestimate parturition date both at the individual- and herd-level. The inclusion of a variance-covariance matrix into the model could control for the lack of independent movement in the MR herd.

In terms of movement behavior the Fogo herd was more similar to the sedentary caribou in DeMars et al. [8] than the MR herd. The event thresholds of the PBM for the Fogo herd were comparable to the thresholds from sedentary caribou in British Columbia (parturition: 23 m/h for Fogo 15 m/h for sedentary from British Columbia; mortality: 126 m/hr for Fogo and 187 m/h for sedentary from British Columbia; [8]). However, this similarity did not appear to lead to improved performance; improved performance could have been masked by low sample size in the Fogo herd (*n* = 9) and the rate at which visual observations occurred (i.e. early mortalities could have been missed by observers).

While the number of calf-cow pairs is comparable between herds, (10 for MR and 9 for Fogo), the proportion of individuals sampled in the MR and Fogo herds differed considerably. We sampled 10 calf-cow pairs and 43 adult females out of approximately 10 000 individuals in the MR herd compared to 9 calf-cow pairs out of approximately 300 individuals in the Fogo herd. This unbalanced sample size could affect the inferences made from the DeMars model. In particular, we make herd-wide inferences about calf survival using < 1% of the herd for MR compared to 3% of the herd for Fogo. However, it is notable that despite small samples sizes offspring survival analyses generated from the IBM and PBM on GPS collared adult females was comparable to survival analyses derived from calf VHF collars. Additionally, there was less variation in parturition and mortality states in the calf-cow pairs (e.g., no non-parturient females in MR, and 2 calves survived to 4 weeks in Fogo), which meant that we were unable to test the performance of the PBM as rigorously.

Even though both the IBM and PBM did not perform as well using migratory woodland caribou in Newfoundland than for sedentary woodland caribou in British Columbia [8], the way in which performance varied among the two methods was similar. Like DeMars et al. [8], we found that the PBM more accurately predicted parturition and the IBM more accurately predicted calf mortality before four weeks. More specifically, DeMars et al. [8] noted that over 95% of incorrect IBM predictions resulted from adult females with surviving calves being misclassified, which is comparable to our IBM where 92% of incorrect IBM predictions resulted from adult females with surviving calves being misclassified as non-parturient cows. It is possible that the start date for the time interval over which we tested (May 21) was too close to actual calving period and thus there was insufficient pre-calving data for the IBM to accurately detect parturition. Unfortunately our collars attempted a GPS fix every two 2 hours from May 21 –July 31 and every five hours for the remainder of the year and therefore an extension of the pre-calving interval was not possible. The strengths of the IBM and PBM offset the weaknesses of each method, presenting us with an opportunity to combine both methods to synthesize best-case results. When parturition was predicted using PBM and calf mortality before four weeks was predicted using IBM in the migratory MR herd, the resulting herd-wide estimates of calf survival were not different from those generated using traditional survival analysis of VHF-collared calves (Fig 4).

When a method accurately predicted an event (PBM for parturition and IBM for calf mortality), the precision around date of occurrence was either low (IBM for calf mortality) or consistently biased (PBM for parturition). This imprecision was detectable at both the individual- and herd-level. At the herd-level we compared our IBM and PBM predicted event date distributions to distributions from VHF-collared calves. These distributions have their own limitations, for example parturition dates (as indexed by collaring dates) might not be representative of the entire herd because researchers generally collar animals during a few days of the calving season on any given year due to logistics [13]. This non-representative sampling could have an obvious effect on generating distribution of parturition dates but could also influence the distribution of calf mortality dates, as parturition date influences calf mortality risk in caribou [13]. If precision in predicting event date using the IBM and PBM methods can be improved, they would represent non-biased herd-wide distributions of these events, which in turn could improve survival analysis using VHF-collared calves.

## Conclusions

A movement-based approach to ecology and wildlife research represents a way to actively and retroactively collect important data on fitness measures, such as parturition and neonate survival, eliminating the need for techniques that may be invasive to vulnerable demographics (i.e., neonates). Ungulate conservation necessitates an understanding of reproduction and survival of juveniles to comprehend the implications on population dynamics [18]. The DeMars model represents an elegant application of movement ecology that may ultimately lead to effective remote quantification of parturition and neonate mortality, thereby adding yet another measure of an important vital rate to a manager’s toolbox. Specifically, it allows for a more effective use of scarce financial and human resources, by allowing multiple analyses and study objectives to be derived from the same telemetry dataset. GPS monitoring while assessing neonate survival with the DeMars model may provide a meaningful and financially feasible alternative to monitoring the herd should a population’s decline accelerate. By collaring adult females (and using the DeMars model) managers and researchers can assess not only adult survival, habitat use, and spatial ecology, but also neonate survival—a vital demographic rate and fitness correlate for some ungulates [18,33].

The DeMars model has potential to be broadly applicable. For migratory woodland caribou in Newfoundland, the IBM accurately predicted calf mortality but not parturition and the PBM accurately predicted parturition but not calf mortality. Where the DeMars model did not perform as well in our system could be related to violations in the assumption of independent movement, due to the behavior of the migratory herd, or constraints in data quality or time interval over which the data were assessed. This presents a problem for the transferability of this model to other ungulate species, other caribou ecotypes, or even caribou populations that exhibit variation in movement behavior different from those studied by DeMars et al. [8]. Furthermore, the variation in event threshold was greater between herds than within, which means the PBM will be more accurate when event thresholds are generated for each population or herd with distinct movement behavior. Despite the limitations in our data, by synthesizing the two methods to produce composite results, the DeMars model performed well with migratory woodland caribou in Newfoundland. Thus, if wildlife managers and researchers have a method for validating the DeMars model within their species and system, the DeMars model may be used to make successful inferences on parturition and calf mortality despite violating its assumptions.

## Supporting information

S1 Appendix Supplementary figures and tables. (PDF)

S2 Appendix R code for population-based method. (PDF)

S3 Appendix 4-hour GPS interval data. (PDF)

S1 Supplementary Data (ZIP)
