# Supplementary material for: Implementing a novel movement-based approach to inferring parturition and neonate caribou calf survival
Source: PLoS One. 2018 Feb 21;13(2):e0192204. doi: 10.1371/journal.pone.0192204 (PMC5821316; doi:10.1371/journal.pone.0192204)
Supplement: S1 Appendix — (PDF) [file pone.0192204.s001.pdf]

S1 Appendix. Supplementary figures and tables

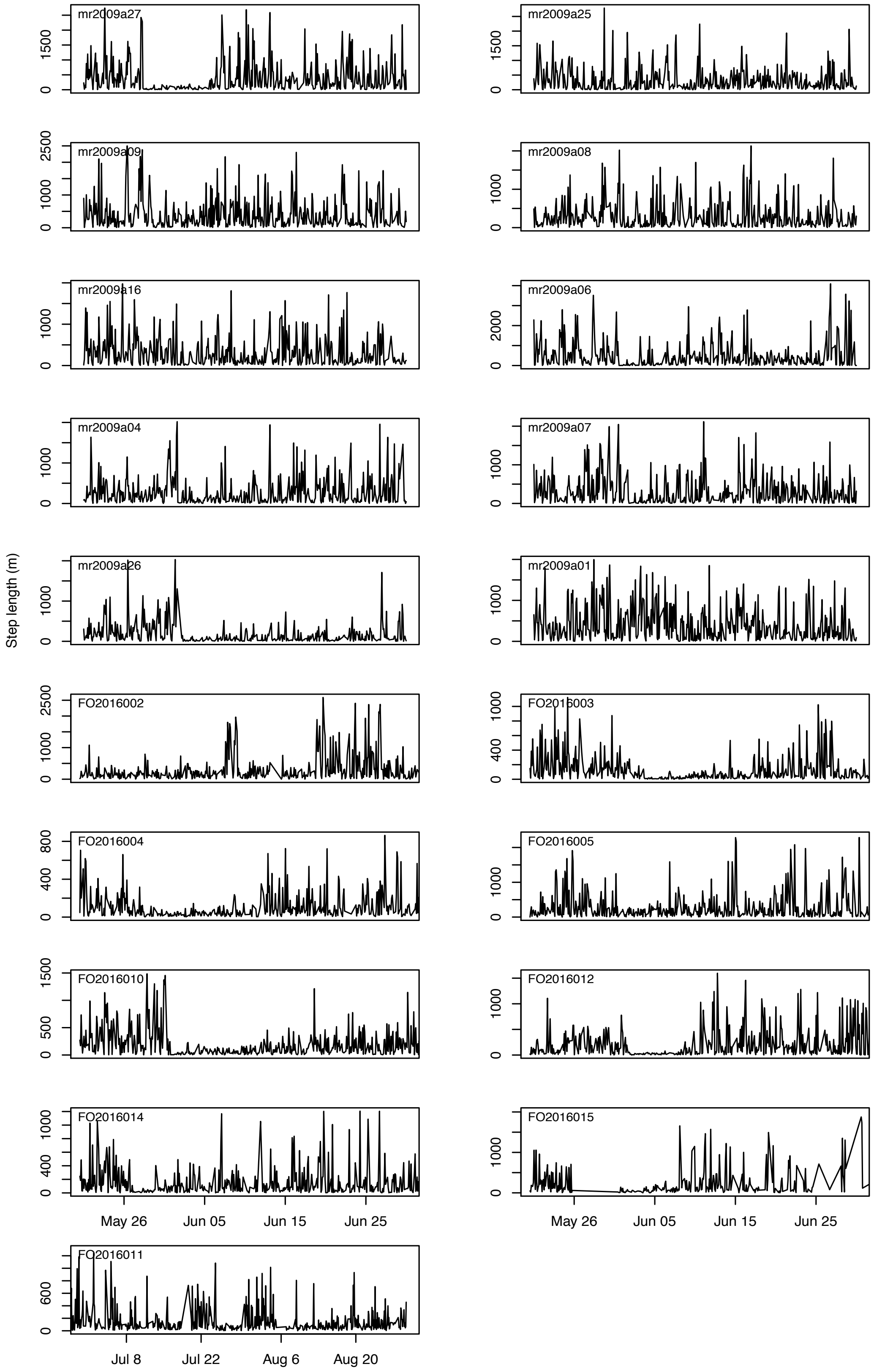

S1 Fig 1. Time-series of step lengths for the 19 calf-cow pairs from Middle Ridge herd and Fogo Island herd. We tested our methods over the time interval 21 May – 30 July for every individual in the Middle Ridge herd and 8 individuals in the Fogo Island herd. We tested our methods over the time interval 30 May – 30 August for one individual to account for a record-late birth in the Fogo herd in 2016 [1]**.**

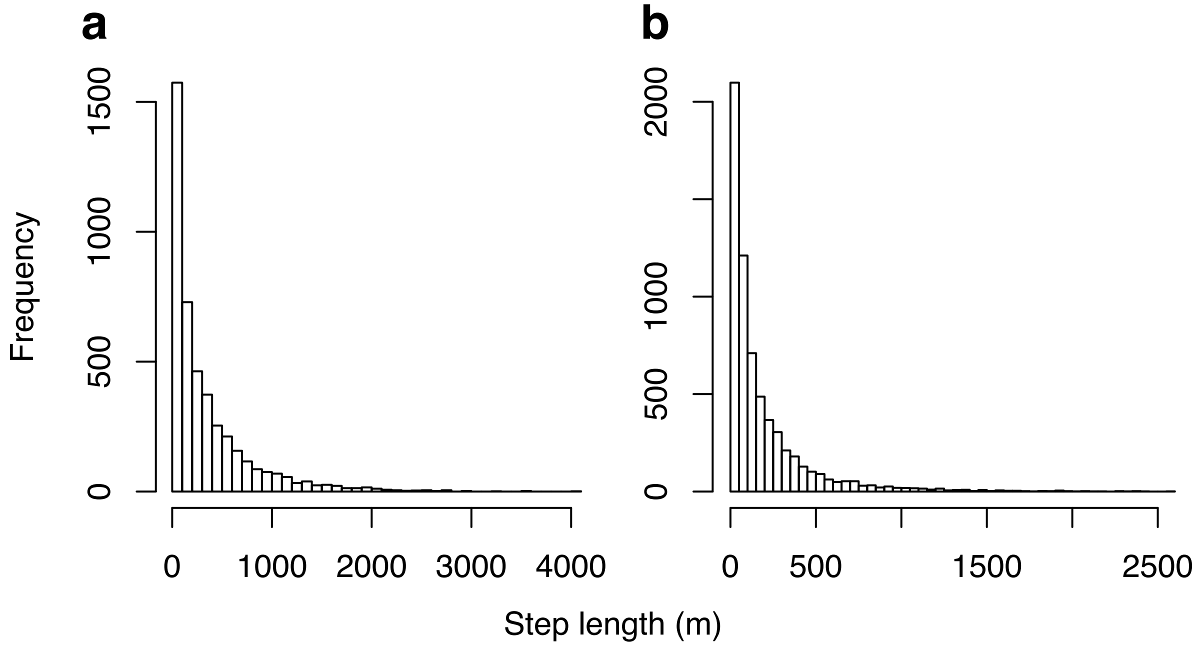

S1 Fig 2. Distribution of step lengths for the 10 calf-cow pairs from Middle Ridge herd (a) and 9 calf-cow pairs from Fogo Island herd (b). Both herds fit the assumption of exponentially distributed step lengths [2]**.**

S1 Table 1. Parturition and calf mortality predictions generated by the population-based method (PBM) from DeMars et al. [2] **for 19 calf-cow pairs from Middle Ridge and Fogo Island herds. The predictions were generated by iteratively sampling of 5 out of the 9 females that could be used to generate the calving and calf loss thresholds for the model and testing on the remaining 14 individuals for all possible combinations. Predictions were pooled and the proportion of each prediction was calculated for every individual.**

	Known Status		PBM Predictions (proportion of time each status was predicted)			
**ID**	**Parturition**	**Calf Survival**	**Parturition**	**No Parturition**	**Calf Mortality**	**Calf Survived**
MR2009a01	Parturition	Survived	1.00	0.00	1.00	0.00
MR2009a04	Parturition	Survived	1.00	0.00	0.00	1.00
MR2009a06	Parturition	Mortality	1.00	0.00	0.50	0.50
MR2009a07	Parturition	Mortality	1.00	0.00	0.50	0.50
MR2009a08	Parturition	Survived	1.00	0.00	0.08	0.92
MR2009a09	Parturition	Survived	1.00	0.00	0.47	0.53
MR2009a16	Parturition	Survived	1.00	0.00	0.07	0.93
MR2009a25	Parturition	Survived	1.00	0.00	0.38	0.63
MR2009a26	Parturition	Survived	1.00	0.00	0.01	0.99

MR2009a27	Parturition	Mortality	1.00	0.00	0.50	0.50
FO2016002	Parturition	Mortality	1.00	0.00	0.44	0.56
FO2016003	Parturition	Survived	1.00	0.00	0.00	1.00
FO2016004	Parturition	Survived	1.00	0.00	0.00	1.00
FO2016005	No Parturition	NA	1.00	0.00	0.05	0.95
FO2016010	Parturition	Mortality	1.00	0.00	0.02	0.98
FO2016011	Parturition	Survived	1.00	0.00	0.00	1.00
FO2016012	Parturition	Mortality	1.00	0.00	0.00	1.00
FO2016014	Parturition	Mortality	1.00	0.00	0.00	1.00
FO2016015	No Parturition	NA	1.00	0.00	1.00	0.00

[1]Individual IDs beginning with MR are from Middle Ridge herd and individual IDs beginning with FO are from Fogo Island herd.

S1 Table 2. Parturition and mortality dates predicted from the individual-based method (IBM) and population-based method (PBM) developed by DeMars et al. [2] **for 43 GPS-collared females in Middle Ridge from 2009−2013. Individuals predicted to have had a parturition event by either or both models are included.**

		IBM		PBM	
**Animal ID**	**Year**	**Parturition date**	**Mortality date**	**Parturition date**	**Mortality date**
MR2009a01	2009			09-05-22	09-05-29
MR2009a02	2009			09-05-22	09-06-03
MR2009a03	2009	09-06-06	09-06-18	09-05-22	09-05-29
MR2009a04	2009			09-05-22	
MR2009a06	2009	09-05-31	09-06-09	09-05-31	09-06-12
MR2009a07	2009	09-06-02	09-06-05	09-05-22	
MR2009a08	2009			09-05-22	
MR2009a09	2009			09-05-23	09-05-27
MR2009a11	2009			09-05-22	09-06-07
MR2009a13	2009			09-05-22	09-06-06
MR2009a14	2009	09-06-18	09-06-03	09-05-22	
MR2009a15	2009			09-05-22	
MR2009a16	2009			09-05-22	
MR2009a17	2009	09-06-23	09-06-28	09-05-22	09-06-02
MR2009a18	2009			09-05-22	09-05-28
MR2009a21	2009			09-05-23	09-05-30
MR2009a23	2009			09-05-22	
MR2009a24	2009			09-05-22	09-06-13
MR2009a25	2009			09-05-23	
MR2009a26	2009		09-06-28	09-05-22	
MR2009a27	2009	09-05-28	09-06-06	09-05-28	09-06-10
MR2009a02	2010	10-06-02	10-06-17	10-05-24	10-05-27

MR2009a03	2010			10-05-22	10-05-29
MR2009a04	2010			10-05-22	10-05-30
MR2009a06	2010			10-05-22	
MR2009a07	2010	10-05-29	10-06-19	10-05-22	
MR2009a08	2010	10-05-28	10-06-01	10-05-22	10-05-24
MR2009a09	2010		10-06-28	10-05-31	
MR2009a11	2010			10-05-22	
MR2009a14	2010			10-05-22	10-06-05
MR2009a15	2010	10-06-04	10-06-19	10-05-22	10-05-30
MR2009a16	2010			10-05-22	
MR2009a17	2010	10-06-08	10-06-11	10-05-22	10-06-05
MR2009a18	2010			10-05-26	10-05-29
MR2009a21	2010			10-06-18	10-06-20
MR2009a24	2010	10-06-16	10-06-28	10-05-22	10-06-10
MR2009a25	2010	10-06-15	10-06-26	10-05-22	
MR2009a26	2010			10-05-22	
MR2009a27	2010			10-05-22	10-05-28
MR2009a28	2010	10-05-23		10-05-24	10-06-02
MR2009a29	2010			10-05-29	10-06-19
MR2009a30	2010	10-06-03	10-06-11	10-06-03	
MR2009a31	2010	10-05-28	10-06-28	10-05-22	10-05-25
MR2009a02	2011			11-05-30	11-06-13
MR2009a03	2011	11-06-04	11-06-18	11-05-23	
MR2009a06	2011			11-05-22	
MR2009a07	2011	11-05-30	11-06-01	11-05-22	11-05-28
MR2009a08	2011	11-06-01	11-06-06	11-05-22	11-05-30
MR2009a09	2011			11-05-28	11-06-13
MR2009a10	2011			11-05-30	11-06-10
MR2009a11	2011			11-05-22	11-06-03
MR2009a14	2011			11-05-22	
MR2009a16	2011			11-05-22	

MR2009a18	2011			11-05-23	
MR2009a21	2011	11-05-29	11-06-02	11-05-23	11-05-27
MR2009a24	2011			11-05-22	
MR2009a25	2011			11-05-24	
MR2009a27	2011			11-05-22	
MR2009a28	2011			11-05-22	
MR2009a30	2011			11-05-22	11-06-01
MR2009a31	2011	11-05-26	11-06-29	11-05-23	
MR2010a01	2011			11-05-22	
MR2010a03	2011			11-05-22	
MR2011a01	2011	11-06-06	11-06-12	11-06-07	11-06-12
MR2011a02	2011	11-05-28	11-06-10	11-05-28	11-06-15
MR2011a03	2011	11-05-27	11-06-10	11-05-28	11-06-11
MR2011a04	2011	11-05-28	11-06-08	11-05-22	
MR2011a05	2011	11-06-13	11-06-15	11-05-22	11-05-30
MR2011a06	2011			11-05-22	11-05-28
MR2009a02	2012	12-05-29	12-06-01	12-05-24	12-06-10
MR2009a03	2012	12-06-07	12-06-29	12-05-22	
MR2009a06	2012			12-05-22	12-05-23
MR2009a07	2012			12-05-22	
MR2009a08	2012			12-05-22	12-05-24
MR2009a09	2012			12-05-25	12-05-27
MR2009a10	2012	12-05-28	12-05-31	12-05-26	12-06-06
MR2009a18	2012	12-06-04	12-06-09	12-05-27	12-05-29
MR2009a21	2012			12-05-22	12-06-11
MR2009a25	2012			12-05-24	
MR2009a30	2012			12-05-25	12-06-15
MR2009a31	2012			12-05-22	12-05-25
MR2010a01	2012			12-05-22	
MR2011a01	2012	12-06-14	12-06-26	12-06-05	12-06-08
MR2011a02	2012			12-05-22	12-05-24

MR2011a04	2012			12-05-25	
MR2009a03	2013		13-06-26	13-05-22	
MR2009a06	2013			13-05-22	13-05-23
MR2009a09	2013	13-05-23	13-06-05	13-05-22	
MR2009a10	2013			13-05-23	13-06-10
MR2009a25	2013	13-05-23	13-05-25	13-05-23	13-05-28
MR2009a31	2013			13-05-22	13-05-23
MR2011a01	2013	13-06-13	13-06-28	13-05-22	13-05-24
MR2011a04	2013	13-05-24	13-05-29	13-05-22	
MR2012a01	2013	13-05-27	13-06-04	13-05-22	
MR2012a02	2013			13-05-22	
MR2012a03	2013			13-05-26	13-06-04
MR2012a04	2013	13-06-03	13-06-06	13-05-22	13-05-28
MR2012a05	2013	13-05-28	13-05-31	13-05-27	13-06-16
MR2012a06	2013	13-05-29	13-06-09	13-05-27	13-06-10
MR 2013a15	2013	13-05-27	13-06-08	13-05-22	

References

1. Bonar M, Laforge MP, Wal E Vander. Observation of a $p < 10^{-9}$ life-history event: implications of record-late caribou birth on ungulate reproductive ecology and field studies. Can J Zool. 2017;95: 133–137.
2. DeMars CA, Auger-Méthé M, Schlägel UE, Boutin S. Inferring parturition and neonate survival from movement patterns of female ungulates: A case study using woodland caribou. Ecol Evol. 2013;3: 4149–4160.
