# Supplementary material for: Implementing a novel movement-based approach to inferring parturition and neonate caribou calf survival
Source: PLoS One. 2018 Feb 21;13(2):e0192204. doi: 10.1371/journal.pone.0192204 (PMC5821316; doi:10.1371/journal.pone.0192204)
Supplement: S2 Appendix — (PDF) [file pone.0192204.s002.pdf]

S2 Appendix. R code for population-based method

Following DeMars et al. [1], our PBM used population level event thresholds (parturition and calf mortality) of 3-day average movement rates (TDAM) in a 3-day moving window analysis to predict calving and calf survival events. To define the parturition threshold, we first generated a distribution of TDAM rates for 3 days post-calving for females who had calves survive to 1 week. We then converted the distribution of movement rates to a kernel density estimate (KDE), which represented the population-level distribution of TDAM rates 3 days post-calving. We transformed this KDE into a cumulative distribution function (CDF) that represented the proportion of the population expected to move at or below this threshold. We then took the 99.9% quantile of the CDF as the parturition threshold; we assumed that movement below this threshold during the moving window analysis indicated calving [1]. To more accurately reflect the true 3-day post-parturition window and thus improve the biological accuracy of the parturition threshold, we estimated parturition date as 1 day prior to calf captures.

We followed the same methods to generate the calf mortality threshold from a distribution of TDAM rates, this time for 2-4 weeks post-parturition for females who had calves survive to 4 weeks old. The 99.9% quantile of CDF from this data represented the maximum TDAM rate of a female with a calf up to four weeks old (i.e., calf mortality threshold); we assumed that movement above this rate indicated calf mortality [1].

Prior to calculating the parturition and calf mortality thresholds, we rarefied the data to exclude the top 1% of step lengths. This removed any step lengths that could have been associated with calf capture or predator avoidance [1]. We generated the parturition and calf mortality thresholds (in the manner described above) in program R [2] using a

function provided by DeMars et al. [1]. We modified the function used to generate the parturition thresholds to reflect the variation in TDAM rates within our data. This is modification was needed because the range over which we calculated the cumulative distribution function was not large enough with the original code. This is likely due to the wide range of movement rates (17m/hr – 340m/hr) within the dataset used to calculate the event thresholds. We needed to increase the range over which the densityFun is created and thus, changed the line `y <- seq(1, max(rollPool)+20, 0.1)` from `+20` to `+200` to account for the wider range of movement rates.

```
makeThresh <- function(moveRates, timeInt, rare=F, draw=F){
      if (rare==T){
        rarIndex <- apply(moveRates, 2, function(x)
    quantile(x, probs=0.99, na.rm=T))
        for (i in 1:ncol(moveRates))
    moveRates[moveRates[,i]  > rarIndex[i],i] <- NA
      }
      rollAverage <- rollapply(moveRates, 3*24/timeInt,
    mean, na.rm=T, by.column=T)
      rollPool <- as.vector(rollAverage)
      rollDensity <- density(rollPool)
      densityFun <- approxfun(rollDensity$x, rollDensity$y,
    yleft=0, yright=0)
      y <- seq(1, max(rollPool)+20, 0.1)
      rollCumu <- rep(NA, length(y))
      for (i in 1:length(y)) rollCumu[i] <-
    integrate(densityFun, -Inf, y[i],
    stop.on.error=F)$value
      quant <- 0.999
      threshold <- y[which(rollCumu >= quant)[1]]
      if (draw==T){
        hist(rollPool, 50, freq=F, xlim=c(0,threshold+10),
    xlab="TDAM mean movement rates", main="Histogram,
    Density and Threshold")
        lines(rollDensity, col='red', lwd=2)
        abline(v=threshold, lwd=2, col='blue')
      }
      return(threshold)
    }
```

```
makeThreshModified <- function(moveRates, timeInt, rare=F,
draw=F){
      if (rare==T){
      if (rare==T){
        rarIndex <- apply(moveRates, 2, function(x)
    quantile(x, probs=0.99, na.rm=T))
        for (i in 1:ncol(moveRates))
    moveRates[moveRates[,i]  > rarIndex[i],i] <- NA
      }
      rollAverage <- rollapply(moveRates, 3*24/timeInt,
    mean, na.rm=T, by.column=T)
      rollPool <- as.vector(rollAverage)
      rollDensity <- density(rollPool)
      densityFun <- approxfun(rollDensity$x, rollDensity$y,
    yleft=0, yright=0)
      y <- seq(1, max(rollPool)+200, 0.1) # Changed from 20
    to 200
      rollCumu <- rep(NA, length(y))
      for (i in 1:length(y)) rollCumu[i] <-
    integrate(densityFun, -Inf, y[i],
    stop.on.error=F)$value
      quant <- 0.999
      threshold <- y[which(rollCumu >= quant)[1]]
      if (draw==T){
        hist(rollPool, 50, freq=F, xlim=c(0,threshold+10),
    xlab="TDAM mean movement rates", main="Histogram,
    Density and Threshold")
        lines(rollDensity, col='red', lwd=2)
        abline(v=threshold, lwd=2, col='blue')
      }
      return(threshold)
    }
```

References

1. DeMars CA, Auger-Méthé M, Schlägel UE, Boutin S. Inferring parturition and neonate survival from movement patterns of female ungulates: A case study using woodland caribou. Ecol Evol. 2013;3: 4149–4160.
2. R Core Team. R: A language and environment for statistical computing. Vienna, Austria: R Foundation for Statistical Computing; 2016. Available: https://www.r-project.org/
