# Supplementary material for: Implementing a novel movement-based approach to inferring parturition and neonate caribou calf survival
Source: PLoS One. 2018 Feb 21;13(2):e0192204. doi: 10.1371/journal.pone.0192204 (PMC5821316; doi:10.1371/journal.pone.0192204)
Supplement: S3 Appendix — (PDF) [file pone.0192204.s003.pdf]

S3 Appendix. 4-hour GPS interval data

Estimating parturition and calf mortality events

DeMars individual-based method

Following DeMars et al. [1], our IBM for parturition and calf mortality events used three *a priori* models representing the three possible states of a female ungulate during calving season: 1) no parturition; 2) calf survived to four weeks old; and 3) calf mortality occurred before the calf was four weeks old (Fig 1). In the model, "no parturition", the mean step length remained constant over time. In the other two models, an event (i.e., parturition or calf mortality) was represented by an abrupt change in the mean step length: a decrease in mean step length represented parturition and an increase represented calf mortality. Thus, in the model, "calf survived to four weeks old", mean step length dropped abruptly, creating a breakpoint at calving, followed by an increase in mean step length with a slope equal to the ratio between the scale parameter and the number of step lengths required for the calf to reach adult movement rates. Conversely, in the model, "calf mortality occurred before four weeks old", mean step length dropped abruptly, creating a break point at parturition, followed by an increase in mean step length with a slope equal to the ratio between the scale parameter and the number of step lengths required for the calf to reach adult rates of movement. This slope, however, was interrupted by an abrupt increase in mean step length to the original mean step length of the adult female at the point of calf mortality [1]. All three of the *a priori* models assumed that step length was exponentially distributed and should differ only in the scale parameter (i.e., mean step length).

We rarefied the datasets to exclude the top 1% of step lengths for each individual, which are thought to be associated with calf capture or predator avoidance behavior [1]. We then generated the IBM for the 10 adult females with known calf status from the MR herd in 2009 and the 9 adult females with known calf status from the Fogo herd in 2016. We present graphical representation of step lengths for all 19 adult females with known calf status in S2 Fig 1. After rarefication, the mean per-collar fix rate (number of successful fixes per number of attempts; [2]) was 83% (range: 47-92%). MR appeared to have a higher mean per-collar fix rate (89%, range: 81-92%) than Fogo (76%, range: 47-85%). Following DeMars et al. [1], we used only step lengths calculated from successive GPS fixes. We used a visual examination of the distributions of step lengths for all 19 calf-cow pairs to determine that the step lengths were exponentially distributed (S2 Fig 2). We then fit the data to each *a priori* model and determined the most supported model using Akaike's Information Criterion (AIC; [3]). We tested our IBM over the time interval 21 May – 30 July for every individual in the MR herd and 8 individuals in the Fogo herd. We tested our IBM over the time interval 30 May – 30 August for one individual to account for a record-late birth in the Fogo herd in 2016 [4]. After applying the IBM to our data, we then compared the predicted calf survival outcomes generated from the IBM with the known outcomes for each female.

DeMars population-based method

The PBM used population level event thresholds (parturition and calf mortality) of 3-day average movement rates (TDAM) in a 3-day moving window analysis to predict calving and calf survival events. Following DeMars et al. [1], to define the parturition threshold, we first generated a distribution of TDAM rates for 3 days post-calving for females who

had calves survive to 1 week. We then converted the distribution of movement rates to a kernel density estimate (KDE), which represented the population-level distribution of TDAM rates 3 days post-calving. We transformed this KDE into a cumulative distribution function (CDF) that represented the proportion of the population expected to move at or below this threshold. We then took the 99.9% quantile of the CDF as the parturition threshold; we assumed that movement below this threshold during the moving window analysis indicated calving [1]. To more accurately reflect the true 3-day post-parturition window and thus improve the biological accuracy of the parturition threshold, we estimated parturition date as 1 day prior to calf captures.

We followed the same methods to generate the calf mortality threshold from a distribution of TDAM rates, this time for 2-4 weeks post-parturition for females who had calves survive to 4 weeks old. The 99.9% quantile of CDF from this data represented the maximum TDAM rate of a female with a calf up to four weeks old (i.e., calf mortality threshold); we assumed that movement above this rate indicated calf mortality [1].

Prior to calculating the parturition and calf mortality thresholds, we rarefied the data to exclude the top 1% of step lengths. This removed any step lengths that could have been associated with calf capture or predator avoidance [1]. We generated the parturition and calf mortality thresholds (in the manner described above) in program R [5] using a function provided by DeMars et al. [1]. We modified the function used to generate the parturition thresholds to reflect the variation in TDAM rates within our data (Appendix C).

We generated PBM estimates for each herd (MR, Fogo) and the combined herds using the 19 adult females with known parturition and calf mortality events. PBM

requires a subset of the population that have experienced both parturition but not calf mortality to generate event thresholds – there was 9 adult females that fit this description (7 in MR and 3 in Fogo). To introduce stochasticity and prevent sampling bias, we iteratively sampled all possible combinations of 5 from the 9 adult females across both herds (*n* = 126) and all possible combinations of 4 from the 7 adult females in MR (*n* = 35; i.e., k-fold) for generating event thresholds. The iterative sampling technique was not possible for the Fogo herd as only 3 of the 9 collared females could be used to calculate the calving and calf loss thresholds. This included the female with the record-late birth outside of the calving season [4], which we chose to exclude since the female may not be representative of the whole herd. Therefore, we had only one estimate of event thresholds for the Fogo herd generated using 2 out of 9 collared females. Using these event thresholds, we then compared the PBM-based predictions of parturition and calf mortality events to the known status of all 19 adult females across both herds and for the MR and Fogo herds separately.

Estimating herd-wide survival, parturition, and mortality date distributions

To generate herd-wide estimates of survival and distributions of parturition and calf mortality dates, we applied both the DeMars IBM and PBM to the 43 GPS-collared adult females from MR. We generated event thresholds using the 7 adult females from the MR herd that had experienced both parturition and calf mortality events. We generated density distributions of the estimated parturition dates and mortality dates from the IBM and the PBM. We also converted the estimated calf parturition and mortality events from the IBM and PBM into Kaplan-Meier survival probability curves using the survival package [6] in R. Following Ellington et al. (in review), we generated herd-wide survival

curves and parturition and mortality date distributions from the 134 VHF-collared calves from the MR herd and compared them to survival curve and distributions generated using the DeMars IBM and the PBM. In all analyses, we generated IBM and PBM models using both a 2-hour GPS fix time interval dataset and a rarified 4-hour GPS fix time interval dataset (to mimic the methods used by [1]).

Results

We present the 4-hour time interval here and have chosen to present the overall results in the body of the manuscript based on the 2-hour time series.

Estimating parturition and calf mortality events

DeMars individual-based method

The DeMars IBM failed to definitively distinguish a parturition and calf mortality status for 3 out of 9 adult females from the Fogo herd (in both cases, the models "calf survived to four weeks old" and "calf mortality occurred before calf was four weeks old" were competing. In one case, parturition occurred, and there was no mortality before four weeks (ΔAIC = 1.22), and in the other two cases parturition occurred and calf mortality occurred before four weeks (ΔAIC = 1.99 and ΔAIC = 1.68). Thus in these cases we considered the IBM method successful in predicting parturition but inconclusive in predicting mortality events. The DeMars IBM definitively distinguished (though not always correctly) a parturition event from a calf mortality event for all 10 adult females from the MR herd.

The IBM correctly classified the two adult females who had no parturition event. The IBM also correctly predicted parturition in 5 of 17 adult female caribou in which parturition occurred (3 of 10 for MR and 5 of 7 for Fogo; S2 Table 1). In situations in which parturition occurred but the IBM method failed to predict parturition (*n* = 9), calf mortality did not occur in 7 of 9 cases (6 of 7 in MR and 1 of 2 in Fogo; S2 Table 1). Indeed, the IBM method correctly identified only 1 of 10 adult females in which parturition occurred but calf mortality did not occur (1 of 7 in MR and 0 of 3 in Fogo; S2 Table 1). The IBM method predicted calf mortality in 3 of 7 adult female caribou in which calf mortality occurred (2 of 3 for MR and 1 of 4 for Fogo; S2 Table 1).

DeMars population-based method

The event thresholds using the PBM were higher in the MR herd than the Fogo herd (parturition: 175 m/hr [range: 117 - 233 m/hr] vs 16 m/hr and calf mortality: 439 m/hr [range: 205 - 585 m/hr] vs 104.1 m/hr). Perhaps this is not surprising given that the dimensions of the island confine space use patterns of caribou in the Fogo herd. The event thresholds using the combined MR and Fogo data were similar to the MR event thresholds, (parturition: 198 m/hr [range: 125 - 257 m/hr] and calf mortality: 426 m/hr [range: 201 - 581 m/hr]).

In general, the PBM performed better for each herd when it used herd-specific event thresholds and when it used event thresholds derived from the combined herds (Table B3), thus we focus our results on PBM based on herd-specific event thresholds. Because of the iterative process in generating event thresholds for the MR herd, the resulting estimates for each event (parturition, no parturition, calf mortality, calf survival) were pooled as proportion of occurrence across all the event thresholds. We considered

the prediction conclusive when the proportion of occurrence was ≥ 0.8 otherwise the prediction was inconclusive. Due to small sample size, there was no iterative process in generating event thresholds for the Fogo herd, thus there were no inconclusive predictions.

The PBM correctly predicted parturition for 16 of 17 females across both herds (for one female in the Fogo herd it predicted parturition when parturition did not occur; Table B2). The PBM did not predict calf mortality correctly and conclusively; in cases where calf mortality occurred the PBM predicted no calf mortality for 3 out of 4 individuals in the Fogo herd (Table B2) and was inconclusive for 2 out of 3 individuals in the MR herd (Table B2). The PBM predicted calf mortality did not occur in 6 of 8 females where calf mortality did not occur (5 of 7 in MR and 1 of 1 in Fogo; Table B2). These results suggested that PBM was more accurate at predicting parturition and the lack of calf mortality than predicting calf mortality, which is opposite to the performance of IBM; the latter was more accurate when predicting parturition with calf mortality than predicting parturition without calf mortality.

Herd-wide survival estimates and distributions of parturition and mortality dates

The predicted distributions of parturition date from the IBM and PBM were different from each other and from the distribution derived from the VHF-collared calves. The IBM predicted that parturition occurred in a wide distribution with only a small peak occurring > 1 week before the observed peak from the VHF-collared calf data, which suggested a long, diffuse calving season (S2 Fig 3a). Conversely, the PBM predicted that parturition occurred in a distribution with a steep peak > 2 weeks before the observed

peak from the VHF-collared calf data, which suggested a calving season broadly similar to the observed calving season but with the majority of parturition events occurring much earlier than they have been observed (S2 Fig 3a). Among individuals with known parturition events, the IBM predicted parturition dates were within 1 day of the collared date (n = 3; i.e., the IBM method when accurate was highly precise; Table B4). The PBM method was highly accurate but predicted parturition dates were typically ≥ 6 days underestimated compared to collar date (n = 8; i.e., the PBM method was highly accurate but had a consistent bias; Table B4)

The predicted distribution of calf mortality dates from the IBM and PBM were broadly similar to the observed distribution from the subset of VHF-collared calves in which mortality occurred prior to 4 weeks of age (S2 Fig 3). The only major discrepancy was that the peak in mortality date occurred slightly later using the PBM than the IBM or the observed VHF-collared calves (S2 Fig 3b). Among individuals with known mortality events (n = 3), the IBM method identified all mortality events but predicted mortality dates varied (-3 to 9 days difference from actual mortality event; i.e., the IBM method was highly accurate but imprecise). The PBM method identified only two of the three known mortality events and consistently overestimated the mortality date (i.e., the PBM method was less accurate and also had a consistent bias).

Both the DeMars IBM and the PBM estimated lower herd-wide survival than what was observed from VHF-collared calves (log-ranked test: $\chi^2 = 17.8$, df = 2, $p < 0.01$; S2 Fig 4a). The IBM estimated 36 parturition events and 25 mortality events, and the PBM estimated 95 parturition events and 4 mortality events. Out of the 134 VHF-collared calves there were 38 mortality events. However, given the performance of the IBM and

PBM when estimating parturition and calf mortality, we generated a survival curve where the PBM was used to identify parturition and the IBM was used to identify mortality (assuming the parturition status identified by PBM). This combined method estimated 104 parturition events and 25 mortality events. Survival rates that we estimated with this combined IBM and PBM method were not statistically different from the 134 VHF collared calves in MR from 2009–2013 (log-ranked test: $\chi^2 = 3.8$, df = 1, p = 0.05; S2 Fig 4b).

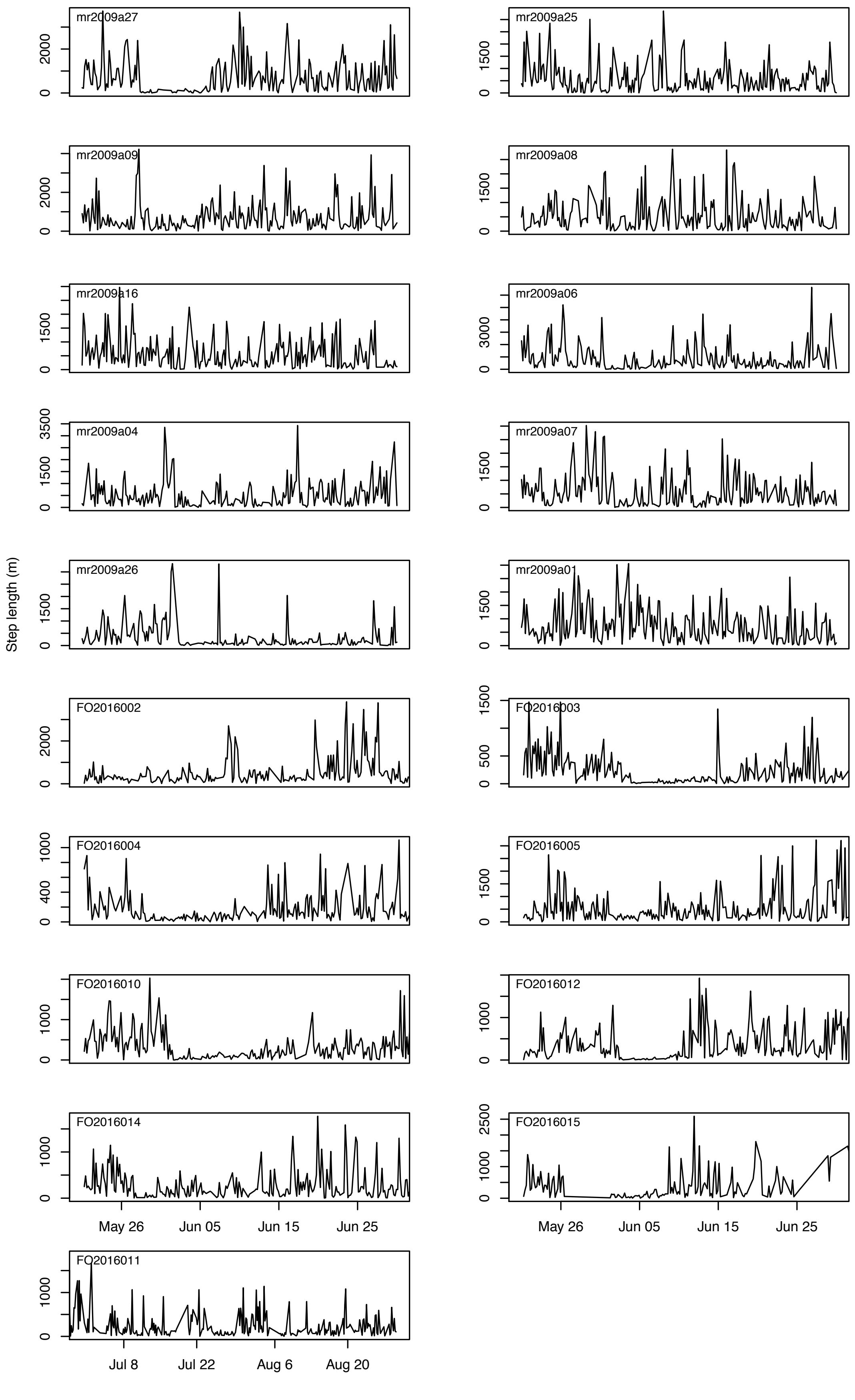
Step length (m)
mr2009a27
mr2009a25
mr2009a09
mr2009a08
mr2009a16
mr2009a06
mr2009a04
mr2009a07
mr2009a26
mr2009a01
FO2016002
FO2016003
FO2016004
FO2016005
FO2016010
FO2016012
FO2016014
FO2016015
FO2016011
May 26
Jun 05
Jun 15
Jun 25
Jul 8
Jul 22
Aug 6
Aug 20

S2 Fig 1. Time-series of step lengths for of the female from the 19 calf-cow pairs from Middle Ridge herd and Fogo Island herd for the 4-hour GPS fix interval. We tested our methods over the time interval 21 May – 30 July for every individual in the Middle Ridge herd and 8 individuals in the Fogo Island herd. We tested our methods over the time interval 30 May – 30 August for one individual to account for a record-late birth in the Fogo herd in 2016 [4].

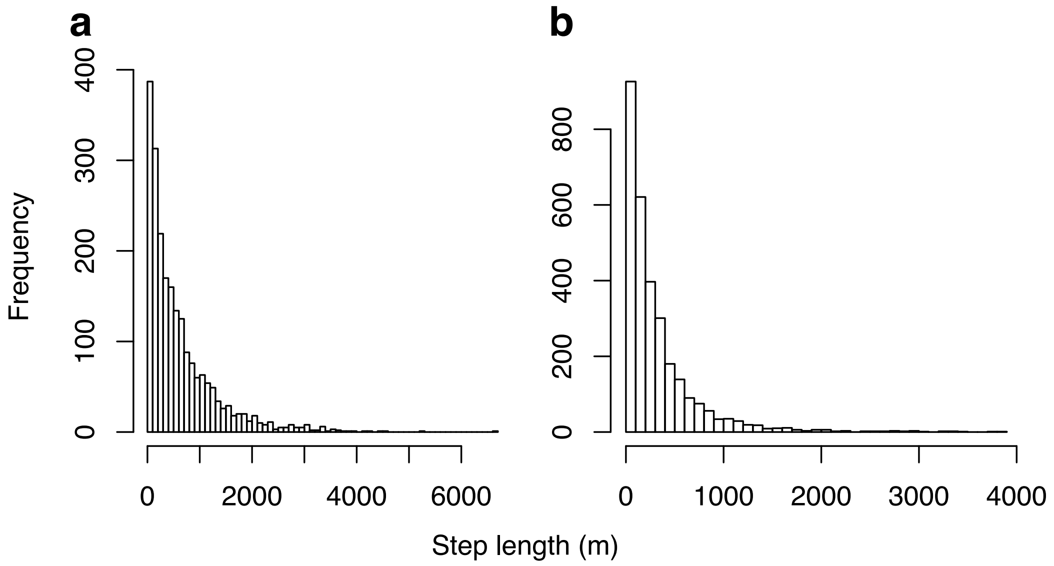

S2 Fig 2. Distribution of step lengths for of the female from the 19 calf-cow pairs from Middle Ridge herd and Fogo Island herd for the 4-hour GPS fix interval. Both herds fit the assumption of exponentially distributed step lengths [1].

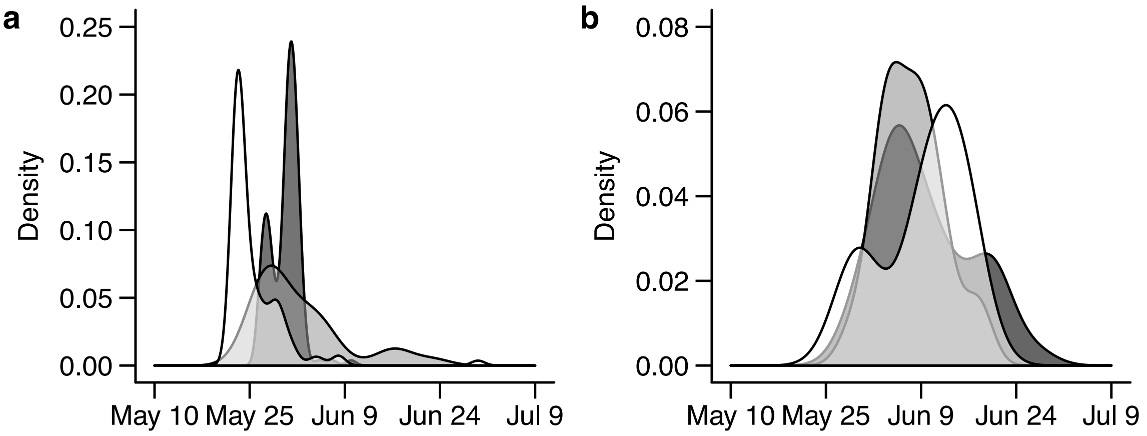

S2 Fig 3. Comparison of density distributions of herd-wide VHF calf collaring dates and mortality dates from [7] with estimated parturition and calf mortality dates derived from DeMars et al. [1] individual-based method (IBM) and population-based method (PBM) at the 4-hour GPS fix interval for 43 adult females from Middle Ridge herd between 2009–2013 (a) Density distributions of herd-wide VHF calf collaring dates (dark gray), estimated parturition dates derived from IBM (light gray) and PBM (white). (b) Density distributions of herd-wide VHF calf mortality dates (dark gray), estimated calf mortality dates derived from IBM (light gray) and PBM (white).

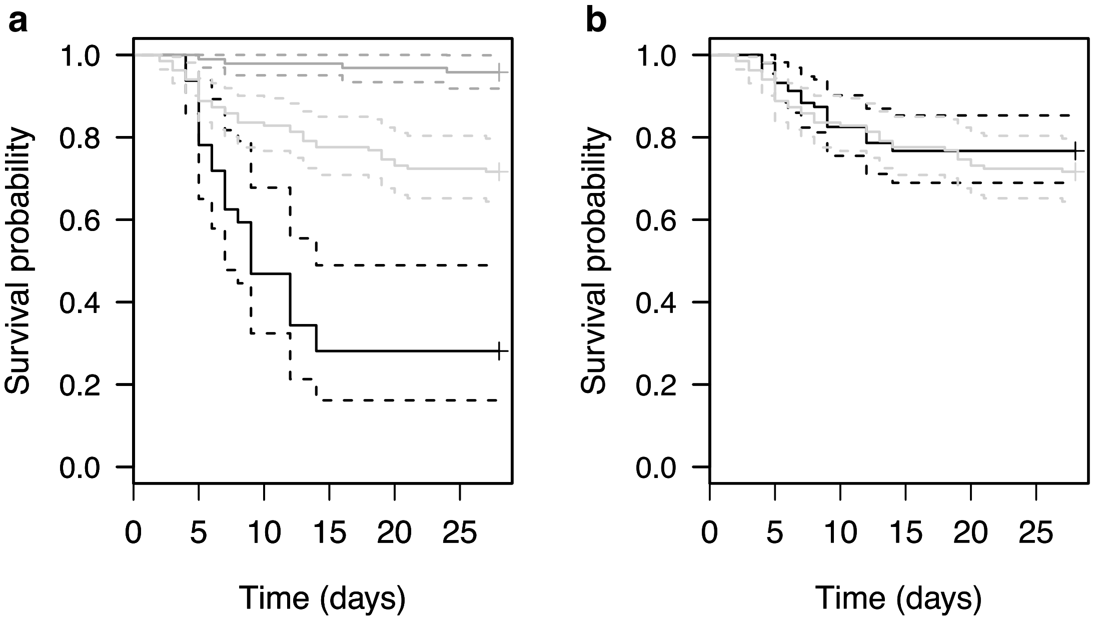

S2 Fig 4. (a) Kaplan-Meier survival probability curves comparing survival data generated at the 4-hour GPS fix interval for 43 GPS-collared females in Middle Ridge from 2009−2013 using DeMars et al.'s [1] individual-based method (IBM; black), and population-based method (PBM; dark gray), and calf mortality from 134 VHF-collared calves in Middle Ridge from 2009−2013 (light gray). Dotted lines are 95% confidence intervals. (b) Kaplan-Meier survival probability curves comparing calf mortality data from 134 VHF-collared calves from Middle Ridge herd from 2009−2013 (light gray) to survival data generated by using a combination of the IBM and PBM models for 43 GPS-collared females in Middle Ridge herd from 2009−2013 (black). Parturition was determined for 43 GPS collared females from 2009–2013 using the PBM and then calf loss was determined using the IBM. For calves whose parturition was predicted using the PBM, if the IBM did not predict calf loss before four weeks we assumed the calf survived.

S2 Table 1. Parturition and calf status predictions derived from the individual-based method (IBM) from DeMars et al. [1] for the 4-hour GPS fix interval. Predictions are for 19 calf-cow pairs from Middle Ridge and Fogo Island herd or which calf status was known.

**Herd**	**Status**	**Observed**	**IBM Predicted**
Middle Ridge	Parturition	10	3
	No Parturition	0	7
	Calf Survival	7	1
	Calf Mortality	3	2
	No Decision	0	0
Fogo Island[1]	Parturition	7	5
	No Parturition	2	4
	Calf Survival	3	0
	Calf Mortality	4	2
	No Decision	0	3

[1] The individual based method for predicting parturition and calf mortality status was inconclusive (competing models) for three adult females

S2 Table 2. Parturition and calf status predictions predicted by the population-based method (PBM) from DeMars et al. [1] for the 4-hour GPS fix interval for 19 calf-cow pairs from Middle Ridge and Fogo Island herd for which calf status was known. Predictions for Middle Ridge herd were generated by iteratively sampling of 4 out of the 7 females that could be used to generate parturition and calf mortality thresholds for the model and tested the on the remaining 6 individuals for all possible combinations. Predictions were pooled and the proportion of each prediction was calculated for every individual. Predictions for Fogo Island herd were generated by using 2 females that could be used to generate parturition and calf mortality thresholds for the model and tested the on the remaining 7 individuals, thus an iterative process was not possible and the 2 individuals used to generate the event thresholds were not included in testing.

	Known Status		PBM Predictions (proportion of time each status was predicted)			
**ID[1]**	**Parturition**	**Calf Survival[2]**	**Parturition**	**No Parturition**	**Calf Mortality**	**Calf Survived[2]**
MR2009a01	Parturition	Survived	1.00	0.00	1.00	0.00
MR2009a04	Parturition	Survived	1.00	0.00	0.07	0.93
MR2009a06	Parturition	Mortality	1.00	0.00	0.43	0.57
MR2009a07	Parturition	Mortality	1.00	0.00	0.14	0.86
MR2009a08	Parturition	Survived	1.00	0.00	0.07	0.93
MR2009a09	Parturition	Survived	1.00	0.00	0.33	0.67

MR2009a16	Parturition	Survived	1.00	0.00	0.00	1.00
MR2009a25	Parturition	Survived	1.00	0.00	0.07	0.93
MR2009a26	Parturition	Survived	1.00	0.00	0.07	0.93
MR2009a27	Parturition	Mortality	1.00	0.00	0.43	0.57
FO2016002	Parturition	Mortality	TRUE	FALSE	TRUE	FALSE
FO2016005	No Parturition		FALSE	TRUE		
FO2016010	Parturition	Mortality	TRUE	FALSE	FALSE	TRUE
FO2016011	Parturition	Survived	TRUE	FALSE	FALSE	TRUE
FO2016012	Parturition	Mortality	TRUE	FALSE	FALSE	TRUE
FO2016014	Parturition	Mortality	TRUE	FALSE	FALSE	TRUE
FO2016015	No Parturition		TRUE	FALSE	TRUE	FALSE

[1]Individual IDs beginning with MR are from Middle Ridge herd and individual IDs beginning with FO are from Fogo Island herd.

[2] When parturition did not occur there was no calf mortality status and when parturition was not predicted there was no calf mortality status predicted.

S2 Table 3. Parturition and calf status predictions predicted by the population-based method (PBM) from DeMars et al. [1] for the 4-hour GPS fix interval for 19 calf-cow pairs from Middle Ridge and Fogo Island herds. The predictions were generated by iteratively sampling of 5 out of the 9 females that could be used to generate the calving and calf loss thresholds for the model and testing on the remaining 14 individuals for all possible combinations. Predictions were pooled and the proportion of each prediction was calculated for every individual.

	**Known Status**		**PBM Predictions (proportion of time each status was predicted)**			
**ID[1]**	**Parturition**	**Calf Survival[2]**	**Parturition**	**No Parturition**	**Calf Mortality**	**Calf Survived**
MR2009a01	Parturition	Survived	1.00	0.00	1.00	0.00
MR2009a04	Parturition	Survived	1.00	0.00	0.11	0.89
MR2009a06	Parturition	Mortality	1.00	0.00	0.46	0.54
MR2009a07	Parturition	Mortality	1.00	0.00	0.17	0.83
MR2009a08	Parturition	Survived	1.00	0.00	0.11	0.89
MR2009a09	Parturition	Survived	1.00	0.00	0.38	0.63
MR2009a16	Parturition	Survived	1.00	0.00	0.00	1.00
MR2009a25	Parturition	Survived	1.00	0.00	0.11	0.89
MR2009a26	Parturition	Survived	1.00	0.00	0.11	0.89

MR2009a27	Parturition	Mortality	1.00	0.00	0.46	0.54
FO2016002	Parturition	Mortality	1.00	0.00	0.17	0.83
FO2016003	Parturition	Survived	1.00	0.00	0.00	1.00
FO2016004	Parturition	Survived	1.00	0.00	0.00	1.00
FO2016005	No Parturition		1.00	0.00	0.17	0.83
FO2016010	Parturition	Mortality	1.00	0.00	0.00	1.00
FO2016011	Parturition	Survived	1.00	0.00	0.00	1.00
FO2016012	Parturition	Mortality	1.00	0.00	0.00	1.00
FO2016014	Parturition	Mortality	1.00	0.00	0.00	1.00
FO2016015	No Parturition		1.00	0.00	0.00	1.00

[1]Individual IDs beginning with MR are from Middle Ridge herd and individual IDs beginning with FO are from Fogo Island herd.

[2] When parturition did not occur there was no calf mortality status.

S2 Table 4. Parturition and mortality dates predicted from the individual-based (IBM) and population-based (PBM) method developed by DeMars et al. [1] for 4-hour GPS fix interval for 43 GPS-collared females in Middle Ridge from 2009−2013. Individuals predicted to have had a parturition event by either or both models are included.

		IBM		PBM	
**ID**	**Year**	**Parturition date**	**Mortality date**	**Parturition date**	**Mortality date**
MR2009a03	2009	09-06-06	09-06-18	09-05-24	
MR2009a06	2009	09-05-31	09-06-09	09-06-01	
MR2009a14	2009	09-05-27	09-06-03	09-05-23	
MR2009a17	2009	09-06-23	09-06-11	09-05-23	
MR2009a26	2009	09-06-01	09-06-25	09-05-23	
MR2009a27	2009	09-05-28	09-06-06	09-05-29	
MR2009a01	2009			09-05-23	
MR2009a02	2009			09-05-23	
MR2009a04	2009			09-05-23	
MR2009a07	2009			09-05-24	
MR2009a08	2009			09-05-23	
MR2009a09	2009			09-05-25	
MR2009a11	2009			09-05-23	
MR2009a13	2009			09-05-23	
MR2009a15	2009			09-05-23	
MR2009a16	2009			09-05-30	
MR2009a18	2009			09-05-23	
MR2009a21	2009			09-05-25	
MR2009a23	2009			09-05-23	

MR2009a24	2009			09-05-23	
MR2009a25	2009			09-05-27	
MR2009a07	2010	10-05-29	10-06-17	10-05-30	
MR2009a09	2010	10-06-01	10-06-13	10-05-29	
MR2009a15	2010	10-06-04	10-06-18	10-05-23	
MR2009a17	2010	10-06-08	10-06-12	10-05-23	
MR2009a24	2010	10-06-17	10-06-26	10-05-23	
MR2009a25	2010	10-06-18		10-05-23	
MR2009a28	2010		10-06-03	10-05-25	
MR2009a30	2010	10-06-03		10-06-05	
MR2009a31	2010	10-05-28		10-05-23	
MR2009a02	2010			10-05-26	
MR2009a03	2010			10-05-26	
MR2009a04	2010			10-05-23	
MR2009a06	2010			10-05-23	
MR2009a08	2010			10-05-23	
MR2009a11	2010			10-05-23	
MR2009a14	2010			10-05-23	10-06-16
MR2009a16	2010			10-05-23	
MR2009a18	2010			10-05-25	
MR2009a21	2010			10-06-30	
MR2009a26	2010			10-05-23	
MR2009a27	2010			10-05-23	
MR2009a29	2010			10-06-04	
MR2009a03	2011	11-06-04	11-06-13	11-05-25	
MR2009a07	2011	11-05-29	11-06-03	11-05-23	

MR2009a08	2011	11-06-01	11-06-06	11-05-23	
MR2009a21	2011	11-05-29	11-06-02	11-05-24	
MR2009a31	2011	11-05-26	11-06-26	11-05-24	
MR2011a01	2011	11-06-06	11-06-13	11-06-08	
MR2011a02	2011	11-05-27	11-06-10	11-05-29	
MR2011a03	2011	11-05-27	11-06-04	11-05-28	
MR2011a04	2011	11-05-27	11-06-08	11-05-23	
MR2011a06	2011	11-05-30	11-06-06	11-05-23	
MR2009a02	2011			11-06-01	
MR2009a06	2011			11-05-23	
MR2009a09	2011			11-05-29	
MR2009a10	2011			11-05-31	
MR2009a11	2011			11-05-23	
MR2009a14	2011			11-05-23	
MR2009a16	2011			11-05-23	
MR2009a18	2011			11-05-24	
MR2009a24	2011			11-05-23	
MR2009a25	2011			11-05-26	
MR2009a27	2011			11-05-23	
MR2009a28	2011			11-05-23	
MR2009a30	2011			11-05-23	
MR2010a01	2011			11-05-23	
MR2010a03	2011			11-05-23	
MR2011a05	2011			11-05-23	
MR2009a02	2012	12-05-29	12-06-04	12-05-25	
MR2009a03	2012	NA	NA	12-05-27	

MR2009a07	2012	12-06-03	12-06-08	12-05-23	
MR2009a18	2012	12-06-04	12-06-09	12-05-29	
MR2009a28	2012	12-05-25	12-05-31	12-05-25	
MR2011a01	2012	12-06-14	12-06-26	12-06-07	12-06-12
MR2009a06	2012			12-05-23	
MR2009a08	2012			12-05-29	
MR2009a09	2012			12-05-26	
MR2009a10	2012			12-05-27	
MR2009a11	2012			12-05-23	
MR2009a21	2012			12-05-29	
MR2009a25	2012			12-05-25	
MR2009a26	2012			12-05-23	
MR2009a30	2012			12-05-26	
MR2009a31	2012			12-05-23	
MR2010a01	2012			12-05-23	
MR2011a02	2012			12-05-23	12-06-08
MR2011a04	2012			12-05-28	
MR2011a05	2012			12-05-28	
MR2009a03	2013		13-06-25	13-05-23	
MR2011a04	2013	13-05-24	13-05-29	13-05-23	
MR2012a01	2013	13-05-26	13-06-04	13-05-23	
MR2012a06	2013	13-05-29	13-06-10	13-05-27	
MR2013a15	2013	13-05-27	13-06-08	13-05-23	
MR2009a06	2013			13-05-27	
MR2009a09	2013			13-05-23	
MR2009a10	2013			13-05-25	

MR2009a25	2013			13-05-24	
MR2009a31	2013			13-05-23	
MR2011a01	2013			13-05-23	13-05-30
MR2012a02	2013			13-05-23	
MR2012a03	2013			13-05-31	
MR2012a04	2013			13-05-23	
MR2012a05	2013			13-05-29	

References

1. DeMars CA, Auger-Méthé M, Schlägel UE, Boutin S. Inferring parturition and neonate survival from movement patterns of female ungulates: A case study using woodland caribou. Ecol Evol. 2013;3: 4149–4160.

2. Frair JL, Fieberg J, Hebblewhite M, Cagnacci F, Decesare NJ, Pedrotti L. Resolving issues of imprecise and habitat-biased locations in ecological analyses using GPS telemetry data. Philos Trans R Soc B. 2010;365: 2187–2200.

3. Burnham KP, Anderson DR. Model selection and multimodel inference. New York: Springer; 2002.

4. Bonar M, Laforge MP, Wal E Vander. Observation of a $p < 10^{-9}$ life-history event: implications of record-late caribou birth on ungulate reproductive ecology and field studies. Can J Zool. 2017;95: 133–137.

5. R Core Team. R: A language and environment for statistical computing. Vienna, Austria: R Foundation for Statistical Computing; 2016. Available: https://www.r-project.org/

6. Therneau T. A package for Survival Analysis in S. 2015. Available: http://cran.r-project.org/package=survival

7. Ellington EH, Lewis KP, Vander Wal E. (in review). Divergent juvenile survival and recruitment: ecological factors and methodological biases. J Wildl Manage -17-0202. 2017.
